# Evidence That Masking of Synapsis Imperfections Counterbalances Quality Control to Promote Efficient Meiosis

**DOI:** 10.1371/journal.pgen.1003963

**Published:** 2013-12-05

**Authors:** Susanna Mlynarczyk-Evans, Baptiste Roelens, Anne M. Villeneuve

**Affiliations:** Departments of Developmental Biology and Genetics, Stanford University School of Medicine, Stanford, California, United States of America; Imperial College London, United Kingdon

## Abstract

Reduction in ploidy to generate haploid gametes during sexual reproduction is accomplished by the specialized cell division program of meiosis. Pairing between homologous chromosomes and assembly of the synaptonemal complex at their interface (synapsis) represent intermediate steps in the meiotic program that are essential to form crossover recombination-based linkages between homologs, which in turn enable segregation of the homologs to opposite poles at the meiosis I division. Here, we challenge the mechanisms of pairing and synapsis during *C. elegans* meiosis by disrupting the normal 1∶1 correspondence between homologs through karyotype manipulation. Using a combination of cytological tools, including S-phase labeling to specifically identify X chromosome territories in highly synchronous cohorts of nuclei and 3D rendering to visualize meiotic chromosome structures and organization, our analysis of trisomic (triplo-X) and polyploid meiosis provides insight into the principles governing pairing and synapsis and how the meiotic program is “wired” to maximize successful sexual reproduction. We show that chromosomes sort into homologous groups regardless of chromosome number, then preferentially achieve pairwise synapsis during a period of active chromosome mobilization. Further, comparisons of synapsis configurations in triplo-X germ cells that are proficient or defective for initiating recombination suggest a role for recombination in restricting chromosomal interactions to a pairwise state. Increased numbers of homologs prolong markers of the chromosome mobilization phase and/or boost germline apoptosis, consistent with triggering quality control mechanisms that promote resolution of synapsis problems and/or cull meiocytes containing synapsis defects. However, we also uncover evidence for the existence of mechanisms that “mask” defects, thus allowing resumption of prophase progression and survival of germ cells despite some asynapsis. We propose that coupling of saturable masking mechanisms with stringent quality controls maximizes meiotic success by making progression and survival dependent on achieving a level of synapsis sufficient for crossover formation without requiring perfect synapsis.

## Author Summary

Diploid organisms must produce haploid gametes prior to sexual reproduction in order to maintain a constant number of chromosomes from one generation to the next. Ploidy reduction is accomplished during meiosis and requires crossover recombination-based linkages between homologous chromosomes. Here, we manipulate karyotype in *C. elegans* to probe the mechanisms that govern stable, pairwise, homologous associations essential for crossover formation. We find that chromosomes sort into homolog groups regardless of number prior to stabilizing interactions (“synapsing”) in a preferentially pairwise manner. Increased numbers of homologs delay meiotic progression and/or boost cell death, reflecting operation of quality control mechanisms that either buy time to correct synapsis problems or eliminate defective cells. Moreover, we found evidence for mechanisms that can “mask” synapsis imperfections, thus allowing resumption of meiotic progression and survival of germ cells when synapsis is “good enough”, albeit imperfect. This strategy would maximize meiotic success by making progression and survival contingent on achieving a level of synapsis sufficient for crossover formation without imposing an onerous and unnecessary requirement for perfect synapsis. We suggest that the regulatory logic of coupling saturable masking mechanisms with stringent quality controls may be employed widely to maximize efficiency of biological circuits.

## Introduction

Sexually reproducing organisms must undergo reduction in ploidy during gametogenesis in order to maintain a diploid chromosome complement from one generation to the next. Ploidy reduction is accomplished at the first division of meiosis, when homologous chromosomes segregate to opposite spindle poles. Segregation of homologs is enabled by a multi-step program of events during meiotic prophase that culminates in most organisms in the formation of recombination-based linkages (chiasmata) between each chromosome pair. Maturation of recombination intermediates into crossovers that can provide the basis of chiasmata occurs in the context of a transient, meiosis-specific structure known as the synaptonemal complex (SC). The SC is a highly ordered proteinaceous structure that assembles at the interface between paired homologs and stabilizes their alignment (reviewed in [1], [2]). While the SC usually connects homologs along their full lengths, evidence suggests that its components function locally to promote crossovers [3]. The SC then disassembles as bivalent chromosomes joined by chiasmata prepare for segregation.

Assembly of the SC (termed synapsis) between paired homologs takes place early in meiotic prophase during a period of active chromosome movement. In organisms from yeasts to mammals, chromosomes within the nucleus become attached to the cytoskeletal motility apparatus in the cytoplasm via a conserved nuclear envelope (NE)-spanning protein complex [4]. In the *C. elegans* system, attachment occurs at specialized domains located near one end of each chromosome called “pairing centers” (PCs) [5], [6]. PCs are bound by a family of DNA binding proteins that associate, in turn, with the NE-spanning SUN-1/ZYG-12 complex, enabling motor-driven, microtubule-dependent chromosome movements [7]–[13]. In *C. elegans*, this chromosome mobilization is important for efficient and timely homolog pairing and for regulation and/or efficient propagation of synapsis, but continues after both processes are largely complete [11], [14]–[16]. Further, recombination is completed only after mobilization ends at mid-prophase [11]. Thus, timely entry into and exit from the chromosome mobilization phase are both critical for forming crossovers between homologs.

Proper coordination of the events of the meiotic program is essential to a successful outcome. Recent work in *C. elegans* has highlighted the importance of checkpoint-like coupling mechanisms that make progression of the meiotic program contingent upon successful execution of prerequisite events. For example, licensing of SC assembly is coupled to homolog identification through a mechanism that likely acts at the level of the PCs, which locally stabilize homolog pairing independent of synapsis, and also promote synapsis between homologs and/or inhibit non-homologous synapsis [9], [10], [17]–[19]. This coupling is critical because the SC assembles in a cooperative manner, yet is structurally indifferent to homology. Another coupling mechanism makes exit from the chromosome mobilization phase contingent upon successful SC assembly [17], [19]–[21]. Exit is delayed in response to incomplete synapsis, presumably allowing more time to ensure that all chromosome pairs are synapsed, and thus competent to form crossovers, before meiocytes proceed to the next stage of meiotic prophase. Finally, several recent studies suggest that meiocytes also have the capacity to detect the presence or absence of sufficient crossover-competent recombination intermediates *per se*, and to respond by enabling or delaying a major transition affecting multiple distinct aspects of the meiotic program [21]–[23].

Coupling mechanisms represent a class of quality control systems that contribute to meiotic success by promoting steps essential to the formation of crossovers between all chromosomes. A second class of quality control systems is represented by checkpoint mechanisms that prevent meiotic segregation errors by eliminating defective cells prior to completion of meiosis. A recombination/DNA damage checkpoint detects unrepaired recombination intermediates in late meiotic prophase and triggers apoptosis of affected meiocytes [24]. In addition, a synapsis checkpoint can trigger apoptosis in response to synapsis failure *per se* [25]. Thus, coupling mechanisms and checkpoints together exert stringent quality control over the meiotic program to ensure the production of normal haploid gametes.

Our current understanding of homolog pairing and synapsis in *C. elegans*, as well as how these processes are coordinated and monitored by quality control systems, has been derived largely from analysis of mutants that lack components of the meiotic machinery. While this approach has been highly fruitful, we reasoned that new insights could be gained from an alternative approach: manipulation of the substrate upon which the wild-type meiotic machinery operates.

In the current work, we interrogate the processes of homolog pairing and synapsis during *C. elegans* meiosis by altering karyotype to disrupt the normal 1∶1 correspondence between homologous chromosomes in the context of a fully intact meiotic machinery. This analysis provides insights into the principles governing homolog pairing and synapsis, as well as the quality control systems that promote successful meiosis. Moreover, we uncover evidence for the operation of a masking mechanism that can “hide” synapsis imperfections from the monitoring machinery, thereby counterbalancing stringent quality control. As a result, a significant fraction of meiocytes containing minor synapsis imperfections may be able to continue meiotic progression, escape apoptosis, and successfully complete recombination. We propose that this strategy maximizes meiotic success by making progression and survival contingent on formation of sufficient SC to ensure crossover formation without imposing an onerous and unnecessary requirement for perfect synapsis.

## Results

### Chromosomes sort into homolog groups in trisomic and polyploid *C. elegans*

**Figure 1:**
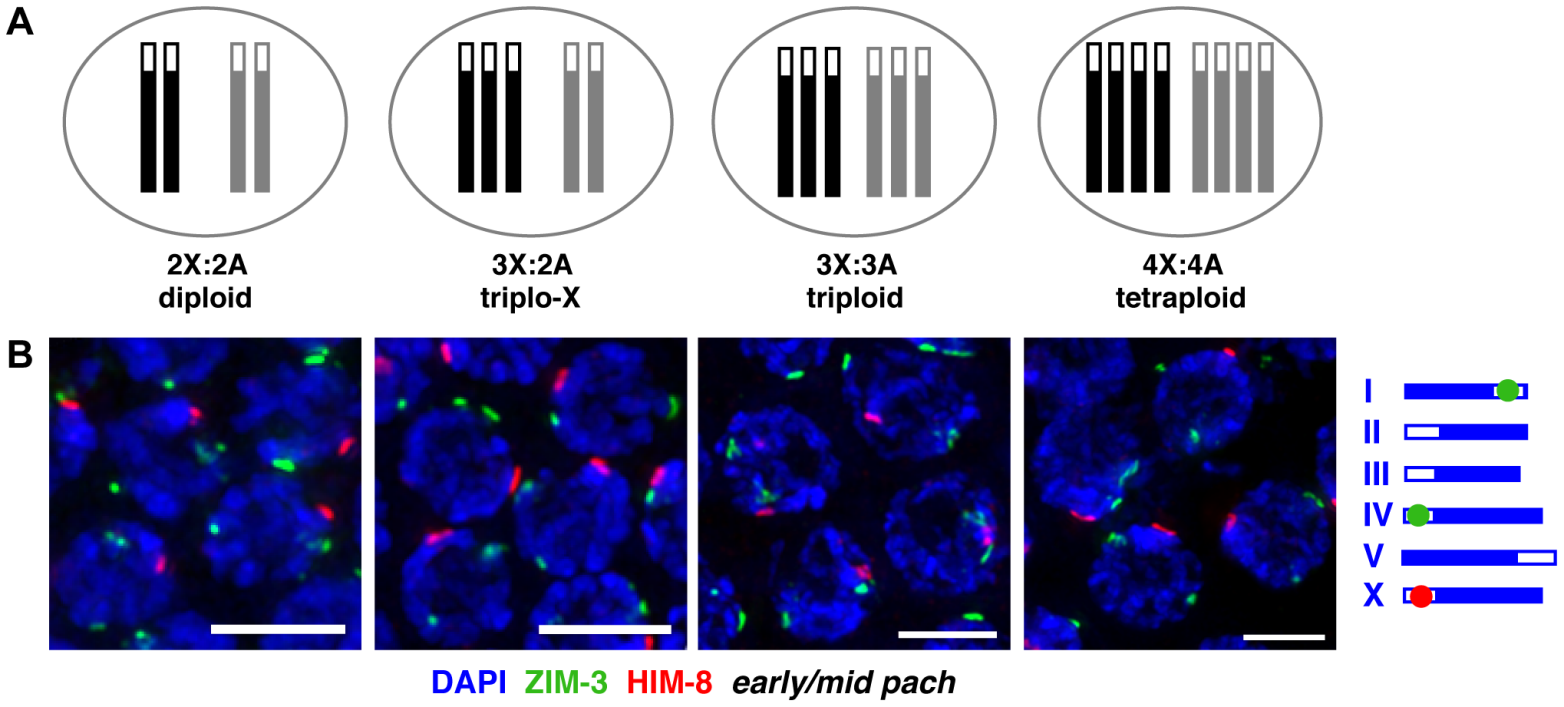
Homolog groups associate at the pairing center in trisomic and polyploid *C. elegans*. **A.** Schematic depictions of the karyotypes of hermaphrodite worms analyzed in this work. X chromosomes are represented in black, autosome sets in gray; open boxes at one end represent PCs. **B.** IF for X-PC binding protein HIM-8 (red) and chromosome I- and IV-PC binding protein ZIM-3 (green, see schematic to right) in karyotypes indicated above; DNA is counterstained with DAPI (blue); full nuclear projections of nuclei in the early pachytene region are shown. One prominent HIM-8 focus and two prominent ZIM-3 foci are typically visible in each nucleus. Minor speckles likely reflect localization of PC proteins to additional chromosomal sites outside of the PC [27], [61] (Figure S1). See Figure S2 for images of full germ lines and quantitation of pairing frequencies at specific time points. Bars = 4 µm.

To gain insight into the mechanisms governing homolog pairing and synapsis, we manipulated the karyotype of *C. elegans* in the context of otherwise wild-type meiotic machinery. We employed hermaphrodite karyotypes containing an increased number of chromosomes, including odd and even numbers of homologs: triplo-X trisomics carrying a third copy of the X chromosome in the presence of a diploid complement of autosomes (3X:2A), full triploids (3X:3A), and full tetraploids (4X:4A; Figure 1A).

We assayed homolog pairing status in trisomic and polyploid *C. elegans* by immunofluorescence (IF) for the PC binding proteins associated with the X chromosome (HIM-8 [8]) and two autosomes (ZIM-3, which binds to the PCs of both chromosomes I and IV [7]). During diploid meiosis, homologous PCs are not associated at meiotic entry; they rapidly pair in the transition zone (TZ, representing the classical leptotene/zygotene stages of meiotic prophase) and remain associated through later stages. Thus, diploid nuclei achieve a single prominent focus of HIM-8 in each nucleus, identifying the paired X-PCs, and two prominent foci of ZIM-3, identifying the separately paired chromosome I- and IV-PCs. In triplo-X, triploid, and tetraploid meioses, we also detected a single prominent HIM-8 focus and two prominent ZIM-3 foci in most nuclei starting in the TZ (Figure 1B, S1, S2) consistent with the ability of groups of two, three, or four homologs to pair at their PCs.

**Figure 2:**
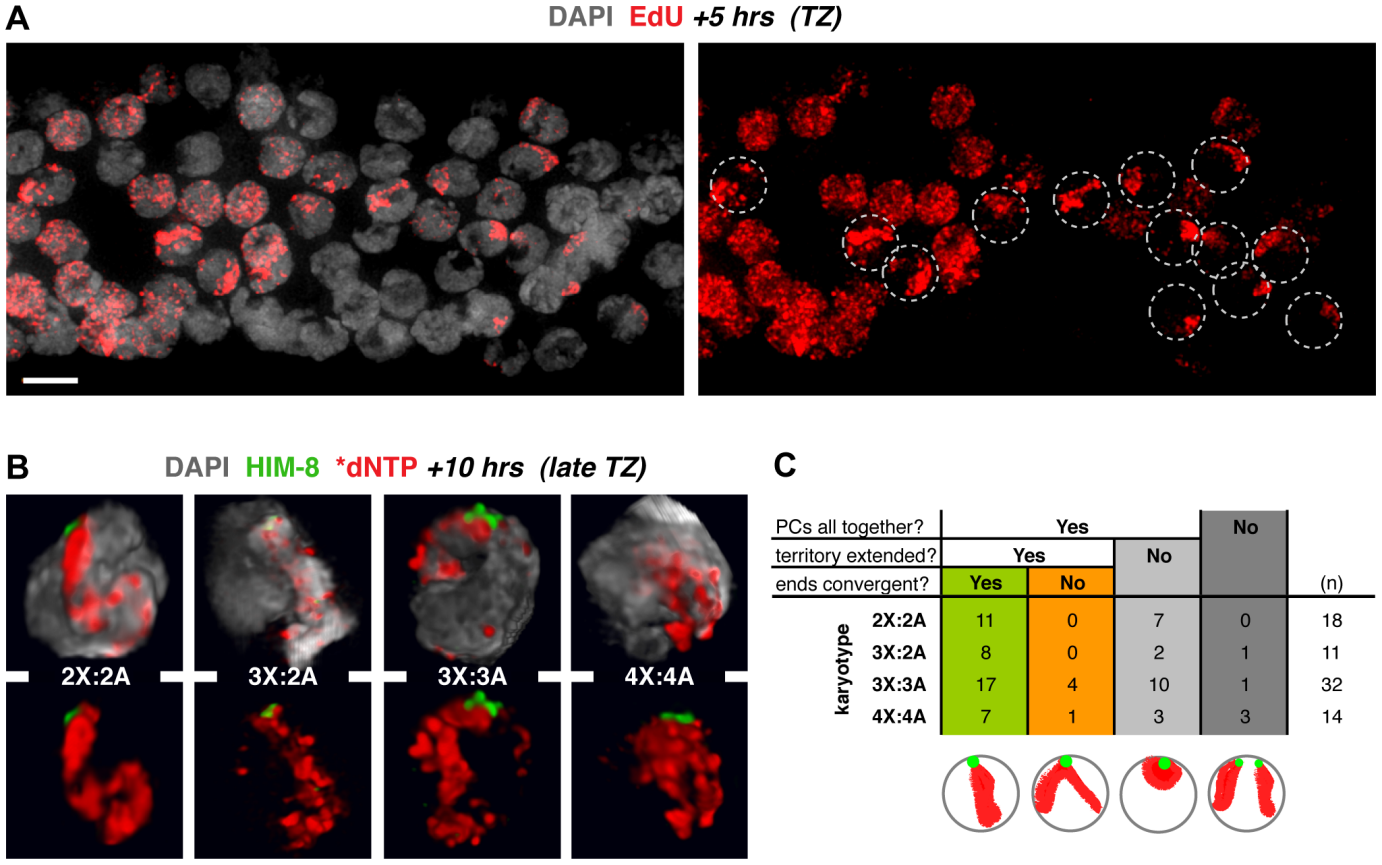
X chromosome territories usually occupy a unitary domain early in the synapsis window. **A.** Use of S-phase labeling to identify a highly synchronous cohort of meiotic prophase nuclei. Image of the TZ region of a diploid germ line (DAPI in gray) labeled with EdU (red) for 5 h prior to fixation; circles indicate synchronous nuclei displaying a single labeled chromosome pair, *i.e.*, the late-replicating X chromosomes. Bar = 4 µm. **B.** 3-D renderings of nuclei early in the synapsis window, illustrating the major class in which PCs are paired and X chromosome territories are “extended” (length greater than twice the width). In all karyotypes the territory comprises a unitary spatial domain suggesting rough alignment. See Figure S3 for all classes. Germ lines were injected with fluorescently labeled dNTPs 10 h prior to fixation and HIM-8 staining (green); nuclei displaying labeled X chromosomes are found in the late TZ in these samples. **C.** Quantitation of the spatial organization of X chromosome territories in nuclei fixed at 10 h post labeling. Categorization is based on X-PC pairing status and labeled X chromosome territory shape as indicated in the schematics. Informative classes are highlighted in green and orange. Nuclei from 4–6 germ lines were analyzed.

To analyze the extent of interactions among groups of three and four homologs at regions beyond the PC, we employed an S-phase labeling approach to identify the nuclear territories occupied by the X chromosomes in a subset of temporally synchronous nuclei. Briefly, live worms were exposed to labeled nucleotide analogs, which incorporate into replicating chromosomes in the germ line. Due to late replication of the sex chromosomes, nucleotides incorporate exclusively into the X chromosome pair in a proximally-located population of S-phase nuclei that are in late meiotic S at the time of exposure [26]. These nuclei, which represent a highly synchronous cohort despite some spatial heterogeneity, can then be analyzed at a desired time point after labeling (Figure 2A).

We used 3-D volume rendering to examine the organization of labeled X chromosome territories in late TZ nuclei, after pairing at the PCs is complete and in the context of ongoing chromosome mobilization (10 h post S-phase labeling; Figure 2B,C; S3). Synapsis is in progress in these nuclei, but is not yet complete. For all karyotypes, the major class of nuclei at this time point displayed paired PCs with X chromosome territories showing an extended appearance. Among diploid nuclei in this class, all showed a unitary labeled domain in which the paths of individual X chromosomes could not be distinguished and non-PC ends appeared to be convergent. This organization suggests close association between the two chromosomes along their lengths, consistent with recent analysis using chromosome paints [27]. Moreover, for karyotypes with three or four copies of the X chromosome, nuclei in this major class typically exhibited characteristics similar to the diploid. Therefore, in addition to pairing at the PC, three and four X chromosomes frequently appear to associate closely along their lengths at this stage, raising the possibility that they may all compete for establishing synapsis interactions.

### Homolog groups preferentially achieve pairwise synapsis, but supernumerary chromosomes challenge this process

**Figure 3:**
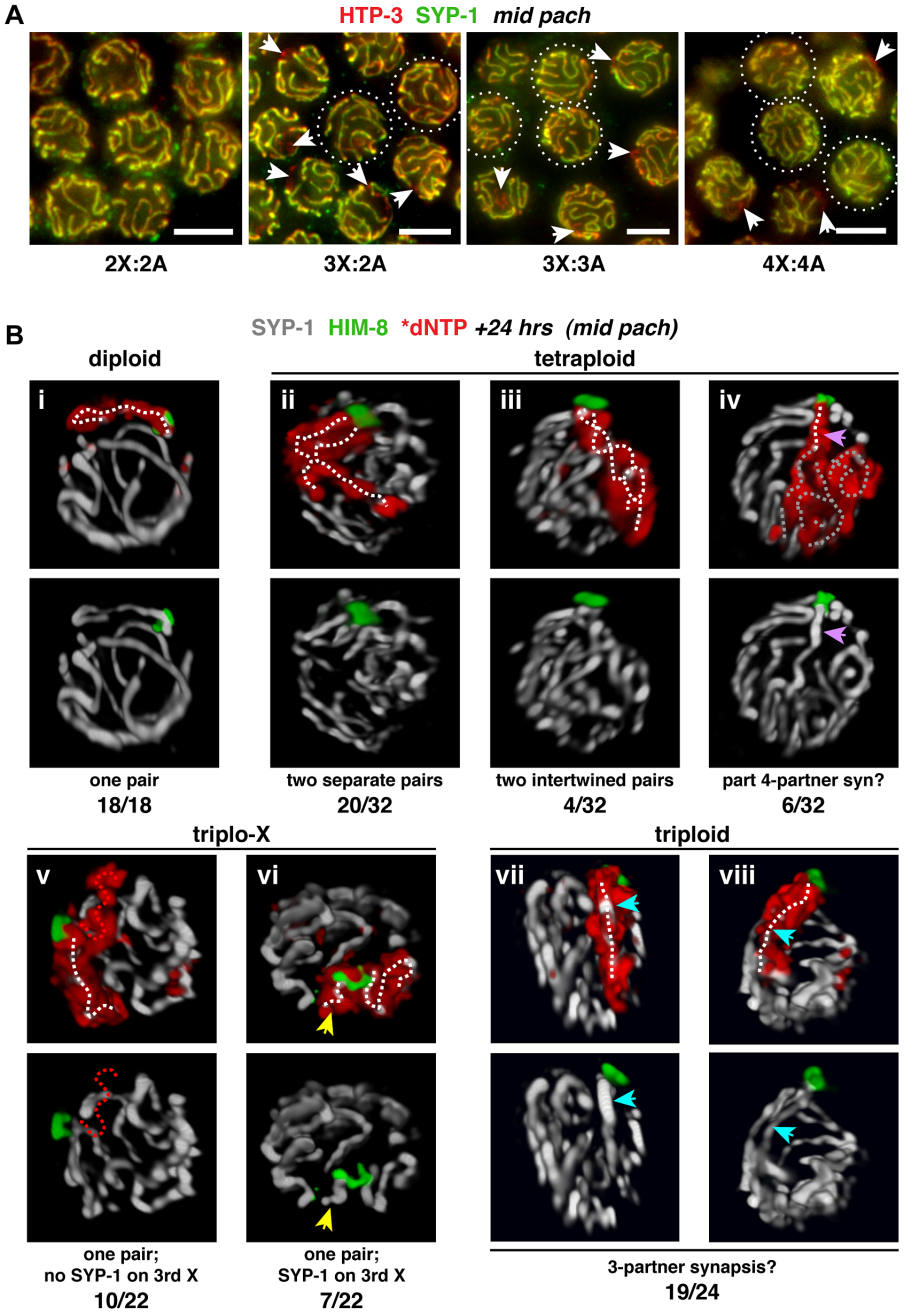
Homolog groups preferentially achieve pairwise synapsis, but supernumerary chromosomes challenge this process. **A.** Synapsis patterns in the indicated karyotypes illustrated by half-nuclear projections of nuclei from the mid-pachytene region. HTP-3 (red) marks chromosome axes and SYP-1 (green) marks SC central region; overlap (yellow) indicates synapsed segments with full synapsis illustrated in the diploid. The altered karyotypes display a mixture of nuclei containing unsynapsed regions (arrows) and nuclei exhibiting apparently complete synapsis (circles; assessments were made from full projections). Images are not deconvolved; bars = 4 µm. **B.** Major classes of X chromosome synapsis configurations in the mid-pachytene region illustrated by 3-D surface renderings of individual nuclei stained for SYP-1 (white) and HIM-8 (green) 24 h after S-phase labeling (red) of X chromosomes. Pairwise synapsis, characteristic of the diploid, also predominates in tetraploid and triplo-X nuclei; whereas in triploids, all three X chromosomes typically occupy a joint domain containing a single SC track. White dotted traces indicate SYP-1 tracks associated with X chromosome territories. iv: region where a single SC track is associated with four X chromosomes (purple arrow), and complex SC tracks associated with the remainder of the X territory (gray dotted traces); v: unsynapsed third X (red dotted trace); vi: short SC track associated with a self-synapsed third X (yellow arrow); vii and viii: single SC track within the domain occupied by all three X chromosomes (blue arrow). Frequency of each synapsis configuration is indicated below each image pair. Nuclei from 4–6 germ lines were analyzed. See Figure S4 for full quantitation of classes in triplo-X and triploids; the remaining two tetraploid nuclei appeared to display partial pairwise homologous synapsis with partial asynapsis or partial heterologous synapsis.

We performed IF to evaluate synapsis patterns in trisomic and polyploid *C. elegans*. Staining for the meiotic chromosome axis protein HTP-3 was used to identify all chromosomes, regardless of synapsis status, and staining for central region protein SYP-1, which bridges pairs of chromosome axes in the mature SC, was used to identify synapsed regions [17], [18]. Examination of mid-pachytene region nuclei, which exhibit full synapsis in the diploid, revealed synapsis aberrations in the altered karyotypes (Figure 3A). While some triplo-X nuclei showed apparently complete synapsis, most displayed a single SYP-1-free region, consistent with an unsynapsed third X in the majority of nuclei. Most triploid nuclei, which contain six odd chromosomes, showed zero to two SYP-1-free regions, indicating that the third copies of each homolog often participate in synapsis interactions. Many tetraploid nuclei showed apparently full synapsis, but some displayed regions lacking SYP-1, suggesting that while full synapsis can be achieved in this karyotype, the process is partially compromised when four copies of each homolog are present. Taken together, these observations indicate that supernumerary chromosomes, whether odd or even in number, present challenges to achieving full synapsis.

To determine the specific synapsis configurations achieved among groups of three and four homologs, we used S-phase labeling in combination with IF and 3-D rendering to trace SC tracks associated with X chromosome territories in synchronous populations of nuclei corresponding to the mid-pachytene stage in diploids (24 h post S-phase). As expected, all diploid nuclei showed a single, robust SYP-1 track associated with the X chromosome pair identified by incorporated label and HIM-8 staining (18/18 nuclei; Figure 3B, i). Synapsis patterns among three and four homologs were more variable, consistent with supernumerary chromosomes presenting challenges to achieving full synapsis; however, analysis of triplo-X and tetraploid nuclei supports the idea that SC tends to connect pairs of chromosome axes.

In the majority of tetraploid nuclei, the X chromosome territories were marked by two SC tracks of similar length emanating from a single HIM-8 focus (24/32 nuclei). Although in most cases the two SCs were separate (Figure 3B, ii), we observed a small class in which they appeared intertwined (Figure 3B, iii); the latter could represent coiling of two separate chromosome pairs around each other or possibly switching of synapsis partners along the lengths of the chromosomes. In addition, a minority of tetraploid nuclei showed a single SC track localizing to the PC end of a domain occupied by all four chromosomes (6/32 nuclei; Figure 3B, iv), suggesting that resolution into pairwise partnerships is partly compromised.

**Figure 4:**
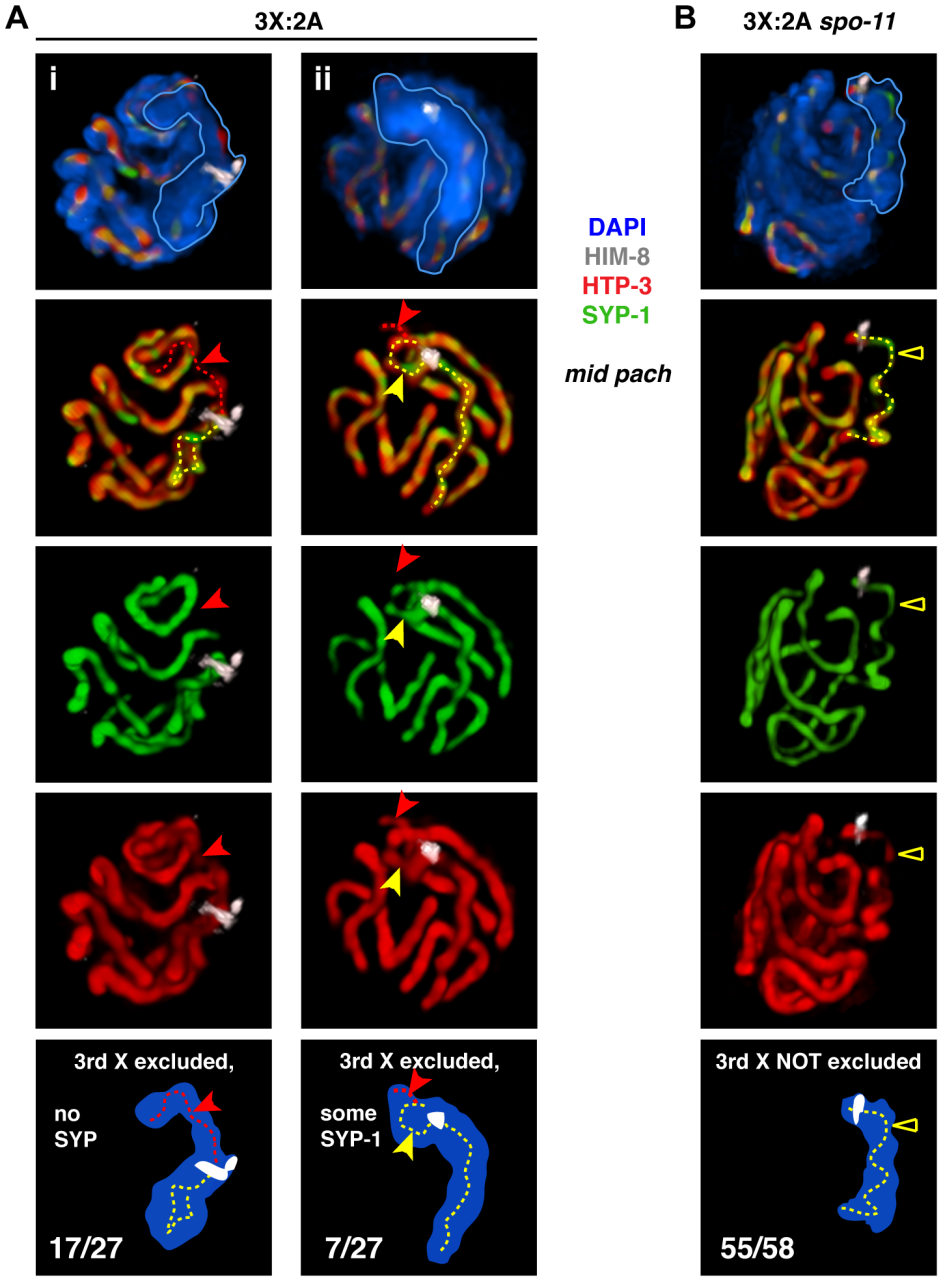
Inability to initiate recombination during triplo-X meiosis impairs establishment of exclusive, pairwise interactions. **A.** Major classes of X chromosome synapsis configurations in triplo-X nuclei illustrated by 3-D surface renderings of individual nuclei stained for HIM-8 (white), HTP-3 (red), SYP-1 (green), and DAPI (blue). Scored nuclei were from the latter half of the region corresponding to mid-pachytene in diploids, which corresponds to the location of S-phase labeled nuclei at the 24 h time point (Figure 3B). Red dotted traces indicate regions of HTP-3 staining where SYP-1 was not detected, and yellow dotted traces indicate regions of HTP-3 and SYP-1 overlap associated with the X chromosomes. Nuclei i and ii both show synapsis between a presumed pair of Xs and exclusion of the third X to a distinct domain of DAPI staining. In i, the third X lacks SYP-1 (red arrow). In ii, the third X shows SYP-1 over part of its axis length (yellow arrow), and absence of SYP-1 over the remainder (red arrow); for clarity, top two-thirds of this nucleus is rendered. Frequency of classes is indicated in the schematics. The remaining three nuclei showed all three X chromosome axes together for part of their length. Nuclei from 3 germ lines were analyzed. **B.** The major class of X chromosome synapsis configuration in triplo-X *spo-11* nuclei, illustrated as in A. The X chromosomes occupy a single, unitary DAPI domain, indicating that the third X is not excluded; the majority of HTP-3 staining within the X chromosome domain appears to localize to a single SC (yellow triangle). The remaining three nuclei showed partial exclusion of the third X chromosome, which showed some SYP-1 loading. Nuclei from 4 germ lines were analyzed.

Our analysis of triplo-X synapsis configurations further supports a preference for pairwise synapsis, extending previous EM analysis of three *C. elegans* triplo-X pachytene nuclei [28]. In the majority of triplo-X nuclei at 24 h post S-phase labeling, a presumed pair of X chromosomes was connected along their lengths by a single SC track, while the third X appeared to be excluded to an adjacent domain (17/22 nuclei). In some cases the excluded third X was SYP-1-negative (10 nuclei; Figure 3B, v), while in others it was SYP-1-positive (7 nuclei; Figure 3B, vi). Finally, in a small number of nuclei all three X chromosomes were found in a unitary territory that displayed a single track of SYP-1, suggesting failure to exclude the third X chromosome in a few cases (5/22 nuclei; Figure S4). All of these categories were detected at comparable frequencies in separate experiments where combined HTP-3 and SYP-1 immunostaining was assessed in mid-pachytene triplo-X nuclei (Figure 4A). These experiments confirmed that when the third X chromosome was spatially excluded, its HTP-3-positive axis either lacked SYP-1 entirely, consistent with asynapsis (Figure 4A, i; Video S1), or showed SYP-1 over part or all of its length, consistent with auto-synapsis that ranged from partial to complete (Figure 4A, ii; Video S2). Taken together our data suggest that by 24 h post-S phase, most triplo-X nuclei have transitioned from initial pairing of all three X chromosomes (characteristic of 10 h post-S phase) to pairwise synapsis accompanied by exclusion of the third X, which is itself sometimes involved in (presumably pairwise) auto-synapsis.

We also examined X chromosome organization in mid-pachytene nuclei from *spo-11* mutant triplo-X worms (3X:2A *spo-11*), which lack the ability to form the DNA double-strand breaks (DSBs) that serve as the initiating events of meiotic recombination. In contrast to otherwise wild-type triplo-X controls, a spatially excluded third X typically was not observed, and a single SC localized to a territory apparently occupied by all three X chromosomes (55/58 mid-pachytene nuclei; Figure 4B; Video S3). Overlap between HTP-3 and SYP-1 signals suggests that this configuration may represent synapsis among three partners (either three-way synapsis throughout, or strictly pairwise synapsis combined with partner switches). Thus, although SPO-11-dependent recombination intermediates are not required in diploids to achieve synapsis between homologs [29], these data suggest that recombinational interactions between chromosomes may nevertheless contribute to maturation of the SC structure into a strictly pairwise state.

Analysis of triploid nuclei by the S-phase labeling method also revealed a defect in establishment of pairwise interactions. While a small minority of triploid nuclei showed apparent exclusion of the third X by 24 h post-S phase (5/24 nuclei, Figure S4), in most cases all three X chromosomes occupied a joint domain to which a single SC localized (19/24 nuclei; Figure 3B, vii and viii). We detected occasional trivalent chromosomes at the diakinesis stage in triploids, indicating that at least a subset of these SCs connect three partners and promote crossovers between them (Figure S5). Together, these observations suggest that the presence of a third copy of every chromosome impairs the ability to solidify pairwise relationships. In addition, we noted a shift toward exclusion of the third X chromosome and emergence of pairwise synapsis in triploids at a later time point (Figure S4). These results raised the possibility that the widespread synapsis challenges in triploids might delay progression through the meiotic program.

### The chromosome mobilization phase is prolonged by challenges to full, pairwise, homologous synapsis

**Figure 5:**
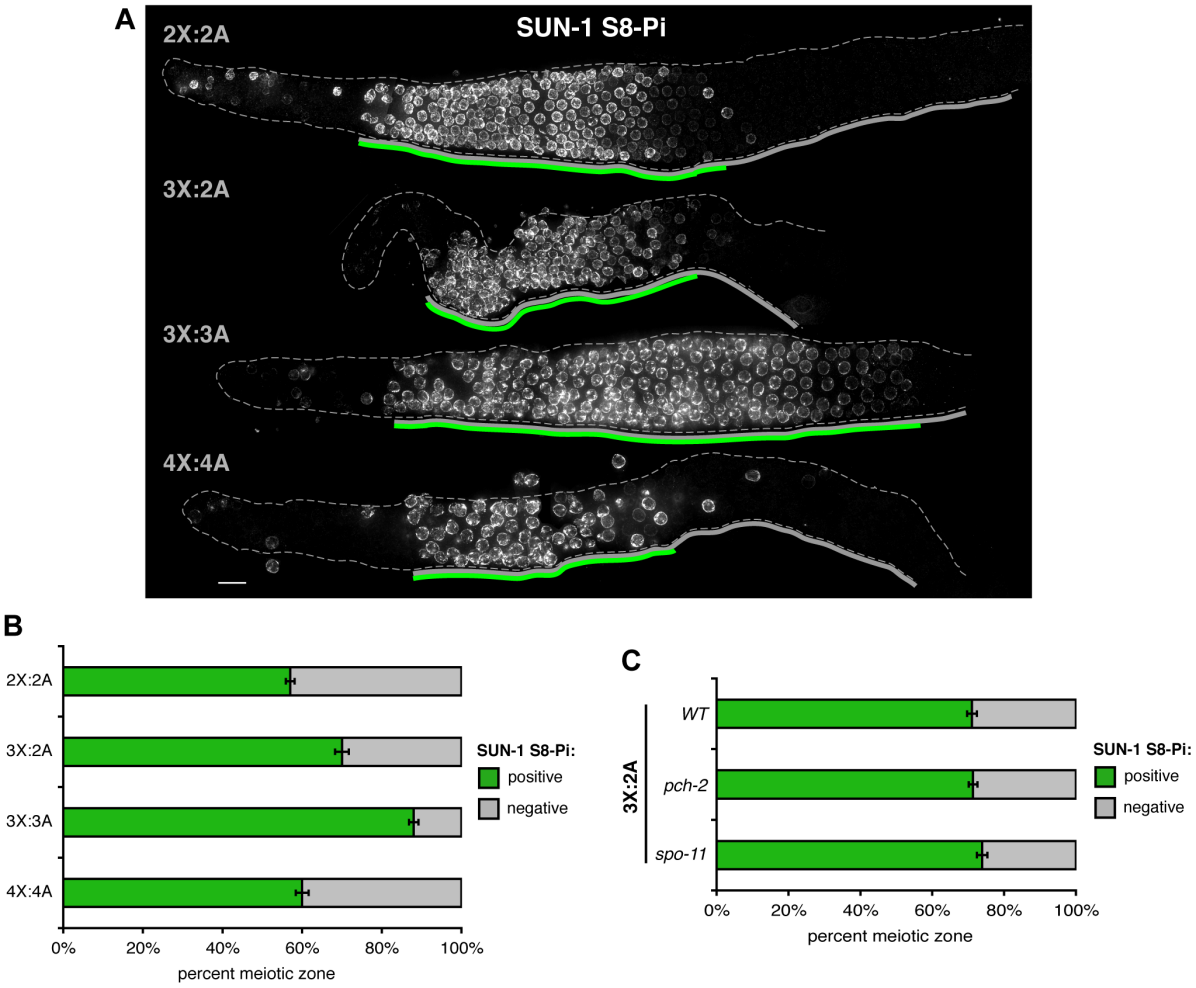
Impact of trisomy and polyploidy on meiotic prophase progression. **A.** Odd numbers of supernumerary chromosomes correlate with a delay in meiotic prophase progression. SUN-1 S8-Pi zones identified by IF for SUN-1 S8-Pi in whole mount germ lines of the indicated karyotypes. Solid gray lines below each germ line indicate the meiotic prophase zone from onset of meiotic SUN-1 S8-Pi staining through the end of the pachytene stage; green lines indicate the SUN-1 S8-Pi zone defined as the contiguous region in which all rows contain 2 or more positively staining nuclei (diffuse NE staining and/or bright NE patches) per row. Bar = 10 µm. **B.** Quantitation of the percent of the meiotic prophase zone occupied by SUN-1 S8-Pi-positive nuclei in each karyotype, scored as illustrated by gray and green lines in A. Data are represented as mean +/− SEM. Numbers of germ lines scored: 2X:2A, 10; 3X:2A, 6; 3X:3A, 14; 4X:4A, 10. **C.** Quantitation of the percent of the meiotic prophase zone occupied by SUN-1 S8-Pi-positive nuclei, scored and represented as in B, in triplo-X germ lines of the indicated genetic backgrounds. Numbers of germ lines scored: wild type, 8; *pch-2*, 15; *spo-11*, 11.

We assessed the impact of supernumerary chromosomes on meiotic prophase progression using IF for a phosphorylated isoform of the NE protein SUN-1 (SUN-1 S8-Pi). In diploid germ lines, SUN-1 S8-Pi is detected in a zone of nuclei extending from TZ entry until the early/mid-pachytene transition [11]. It localizes both diffusely throughout the NE and in concentrated NE-associated patches corresponding to points of attachment between the chromosomes and the cytoskeletal motility apparatus that mediates movement. Moreover, conditions that prolong the duration of chromosome mobilization in diploids extend the SUN-1 S8-Pi zone [11], and this persistent phosphorylation is required to delay meiotic progression [21]. Diffuse SUN-1 S8-Pi is also prolonged in mutants that are proficient for synapsis but impaired in the ability to form crossover recombination intermediates between homologs, but in such cases the multiple bright patches indicative of chromosome movement do not persist [21], [22]. In tetraploid germ lines, the SUN-1 S8-Pi zone occupied a proportion of the meiotic region that was not significantly different from that in diploids (Figure 5A, B; two-tailed P = 0.166, Mann-Whitney Test), suggesting that presence of an even number of supernumerary chromosomes has little impact on the relative length of the chromosome mobilization phase.

By contrast, the two “odd” karyotypes displayed extended SUN-1 S8-Pi zones (Figure 5B, two-tailed P≤0.002, Mann-Whitney Test) with multiple bright patches found in most positively-staining nuclei. Triplo-X germ lines showed a modest extension, with loss of SUN-1 S8-Pi taking place in the region corresponding approximately to the mid-pachytene to late pachytene transition in diploids. Triploid germ lines displayed a more dramatic extension than triplo-X (two-tailed P<0.0001, Mann-Whitney Test), with SUN-1 S8-Pi levels declining only toward the end of the region corresponding to the late pachytene stage in diploids, when constraints on multiple meiotic processes are known to be lifted [19], [30]. These results suggest that odd numbers of supernumerary chromosomes prolong the chromosome mobilization phase and impede meiotic progression. Further, they raise the idea that the meiotic program can ultimately accommodate problems associated with a single partnerless chromosome.

The ability to extend the SUN-1 S8-Pi positive zone in triplo-X germ lines does not require the same machinery as the “synapsis checkpoint” that triggers apoptosis in response to synapsis defects, since the extent of the SUN-1 S8-Pi positive zone is not altered in triplo-X germ lines mutant for *pch-2*, which encodes a conserved AAA+ ATPase required for the synapsis checkpoint (Figure 5C; [25]). Further, the length of the SUN-1 S8-Pi positive zone in triplo-X germ lines was also not altered by mutation of *spo-11*, indicating that that inability to form crossovers between homologs and/or unresolved multi-partner interactions (Figure 4B) do not further prolong the SUN-1 S8-Pi positive state beyond the response elicited by a single extra X chromosome.

### Altered karyotypes are associated with elevated germ cell apoptosis

As defective synapsis and unrepaired recombination intermediates can trigger elevated apoptosis in the late pachytene region of the *C. elegans* germ line through the action of meiotic checkpoints [24], [25], we assessed the impact of supernumerary chromosomes on germ cell apoptosis levels (Figure S6). The outcome of this analysis varied depending on the conditions under which apoptosis was assayed, with significantly elevated numbers of germ cell corpses detected in both triplo-X and triploid (but not tetraploid) gonads under one set of conditions, but not under another. Since the gonads of worms with altered karyotypes contain fewer meiotic nuclei overall (see Figure 5A; Table S1), we also attempted to normalize numbers of germ cell corpses relative to numbers of meiotic zone nuclei (see Figure S6 legend); when normalized values were used, all three modified karyotypes showed a significant elevation of apoptosis compared to diploids, suggesting that meiotic checkpoints may be triggered above baseline levels when supernumerary chromosomes are present. Triploids showed a larger elevation than triplo-X, suggesting a greater degree of checkpoint activation when all chromosomes are present in three copies and providing a parallel to the greater impact on meiotic progression discussed above. However, normalized apoptosis levels in tetraploids were not significantly different from those observed in tripoids despite tetraploids showing relatively normal meiotic progression as assayed by SUN-1 S8-Pi, suggesting that these two features of the meiotic program may not be inherently coupled.

### Efficient acquisition of H3K9me2 by synapsis-challenged chromosomes correlates with ability to exit the chromosome mobilization phase

**Figure 6:**
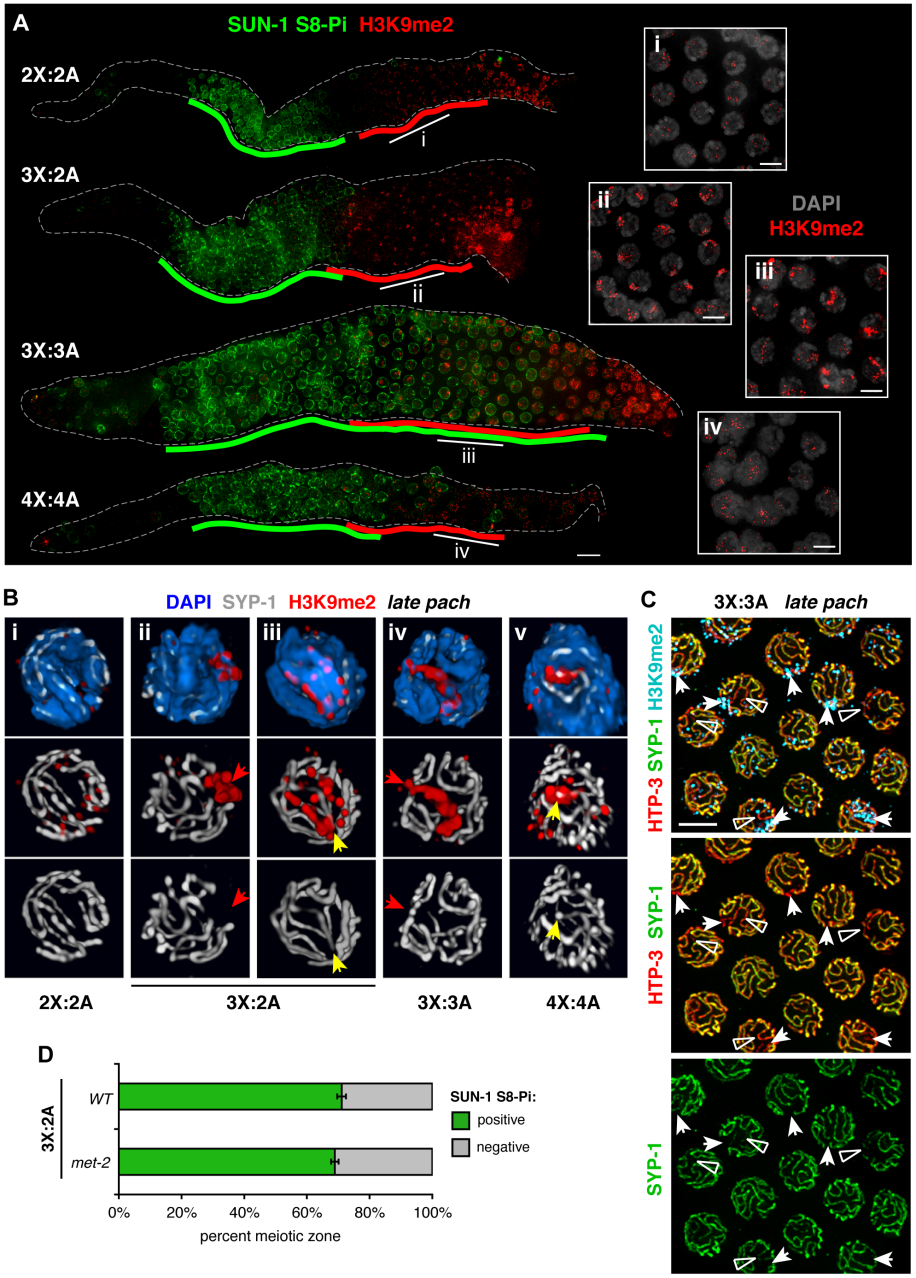
Relationship between H3K9me2 acquisiton at synapsis defects and loss of SUN-1 phosphorylation in altered karyotypes. **A.** Relationship between appearance of the H3K9me2 chromatin mark (red) and downregulation of SUN-1 S8-Pi (green) illustrated by IF in whole mount hermaphrodite germ lines of the indicated karyotypes. Green lines are drawn as in Figure 5, and red lines delineate the region in which the majority of nuclei in each row show the specific mid-prophase nuclear staining pattern characteristic of each karyotype (see text). Bar = 10 µm. Insets (right) show H3K9me2 (red) staining patterns in DAPI-stained nuclei (gray) from indicated regions of the corresponding germ lines. Bars = 4 µm. **B.** Major classes of H3K9me2 (red) and SYP-1 (white) staining observed in late pachytene region nuclei of each karyotype illustrated by 3-D surface renderings; DAPI is shown in blue. In altered karyotypes, intense surface domains of H3K9me2 correspond most commonly to unsynapsed chromatin (red arrows), but sometimes mark synapsed chromatin as well (yellow arrows). **C.** Half-nuclear projection of late pachytene region from a triploid hermaphrodite germ line stained for H3K9me2 (blue), HTP-3 (red) and SYP-1 (green), revealing differential H3K9me2 association with two classes of synapsis defects—regions where SYP-1 staining was undetectable (arrows, usually marked) and where SYP-1 staining was very weak compared to surrounding SCs (open triangles, rarely marked). Preferential association of H3K9me2 with regions where SYP-1 was undetectable was confirmed by analysis of 3-D image stacks. Bar = 4 µm. **D.** Quantitation of the percent of the meiotic prophase zone occupied by SUN-1 S8-Pi-positive nuclei, scored and represented as in Figure 5B and C, in triplo-X germ lines of a *met-2* mutant background (8 germ lines scored). Wild type control is the same as presented in Figure 5C.

In *C. elegans*, unsynapsed chromosomes including the single X in XO males are marked by the histone H3 dimethyl Lys9 (H3K9me2) chromatin modification during the latter half of the pachytene stage [31], [32]. During normal diploid hermaphrodite meiosis, levels of the H3K9me2 mark begin to rise at the early/mid-pachytene transition [33], and we found that this timing coincided approximately with loss of SUN-1 S8-Pi (Figure 6A). H3K9me2 signals commonly appeared faint and scattered (Figure 6B, i) until levels increased dramatically throughout the chromatin at the end of the late pachytene stage. The timing of onset of H3K9me2 staining and the very late prophase rise in H3K9me2 levels were shared by all karyotypes. However, we observed distinct nuclear patterns of H3K9me2 as well as informative relationships to the SUN-1 S8-Pi zone during trisomic and polyploid meiosis.

**Figure 7:**
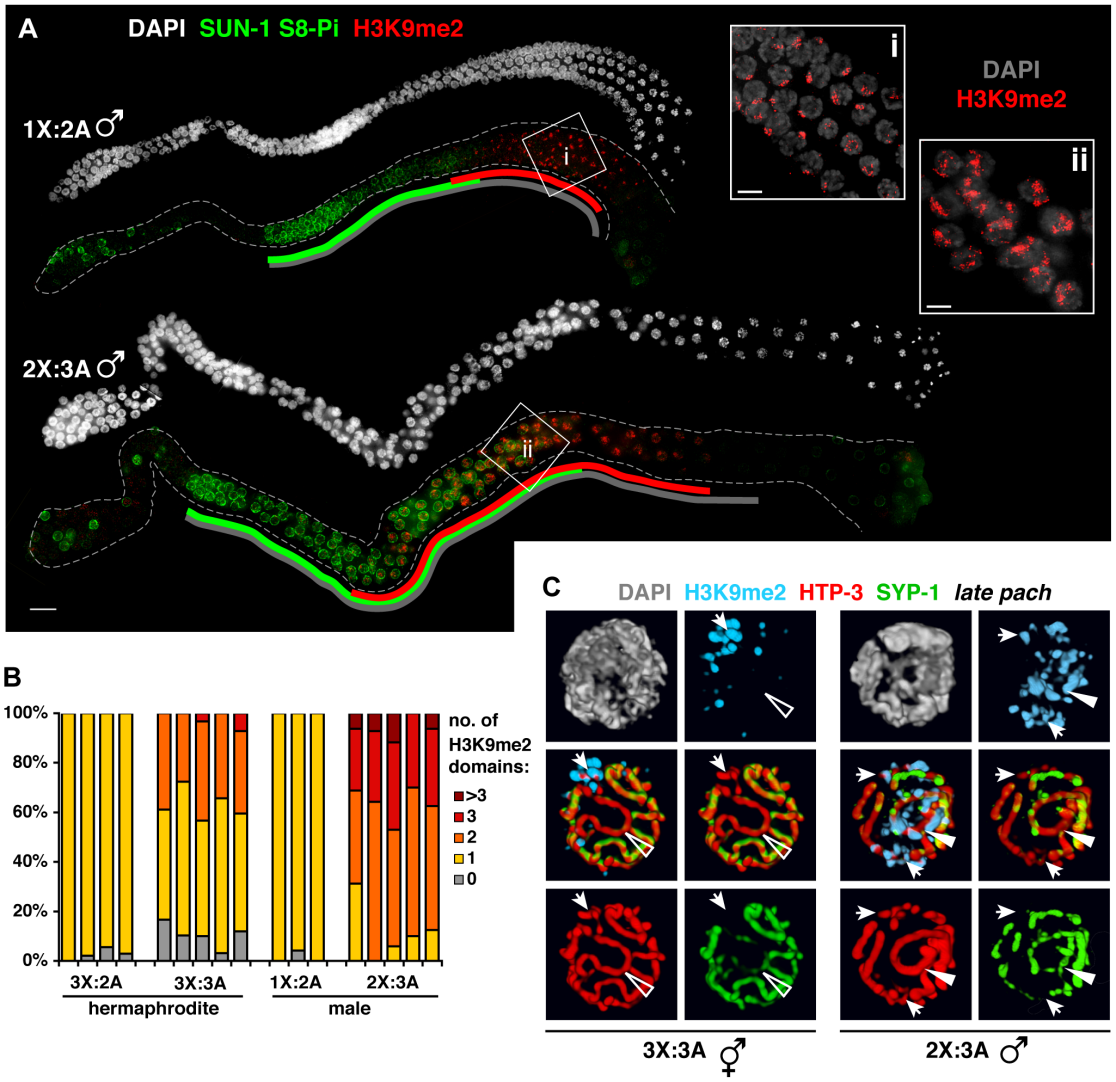
Male germ cells containing supernumerary chromosomes show a greater capacity to apply H3K9me2. **A.** DAPI staining (white) and IF for SUN-1 S8-Pi (green) and H3K9me2 (red) in whole mount male diploid (1X:2A) and triploid (2X:3A) germ lines. Green and red lines are drawn as in Figure 6; gray lines indicate meiotic zone used for scoring in Figure S8, from onset of meiotic SUN-1 S8-Pi staining through the end of the pachytene stage. Bar = 10 µm. Insets (right) show H3K9me2 (red) staining patterns in DAPI-stained mid- to late pachytene nuclei (gray). Bars = 4 µm. **B.** Quantitation of the number of H3K9me2 domains in late pachytene nuclei of hermaphrodite vs. male germ lines with a single odd X chromosome (compare 3X:2A vs. 1X:2A), or a triploid karyotype (compare 3X:3A vs. 2X:3A). 3-D-rendered images were used for scoring. Each stacked bar represents the late pachytene region of a single germ line of the indicated genotype, comprising 18–47 nuclei in hermaphrodites and 14–26 nuclei in males; the “>3” category includes nuclei in which contiguous H3K9me2 domains occupied half or more of the nuclear volume. **C.** Coincidence of H3K9me2 (blue) staining with synapsis defects in triploid hermaphrodites (3X:3A) and males (3X:2A) illustrated by 3-D surface renderings of half-nuclei from the late pachytene region. HTP-3 (red), SYP-1 (green), and DAPI (gray) are shown. Unsynapsed regions (arrows) are marked in both sexes, whereas aberrantly synapsed regions are not marked in the hermaphrodite (open triangle), but robustly marked in the male (filled triangle).

In triplo-X and tetraploid germ lines, acquisition of specific nuclear H3K9me2 patterns correlated with loss of SUN-1 S8-Pi staining (Figure 6A). In the triplo-X, H3K9me2 localized to a single intense domain in every nucleus by the mid-pachytene region (Figure 6A, 7B), consistent with marking of the synapsis-challenged third X chromosome in every nucleus. H3K9me2 domains corresponded to chromatin devoid of SYP-1 in 70% of nuclei (21/30; Figure 6B, ii), and to SYP-1-associated chromatin in 30% of nuclei (9/30; Figure 6B, iii), suggesting that the excluded X chromosome is marked, at least in part, whether or not it loads some SYP-1. In the tetraploid, where synapsis defects are more variable, several H3K9me2 patterns were observed (Figure 6A, iv; 6B, v): some nuclei showed faint signal comparable to the diploid; many showed small intense terminal domains colocalizing with SYP-1; and a few showed large H3K9me2 domains generally corresponding to unsynapsed regions. Together, our data suggest that in trisomic or tetraploid germ cells, chromosomes or chromosomal segments that experienced synapsis challenges are efficiently marked with H3K9me2, whether or not they ultimately acquire SC. Further, co-staining suggests a temporal correlation between appearance of the H3K9me2 mark on synapsis-challenged regions and loss of SUN-1 S8-Pi in these karyotypes.

In the triploid, H3K9me2 domains appeared with similar timing to the other karyotypes, but their appearance was not accompanied by down-regulation of SUN-1 S8-Pi (Figure 6A). Moreover, although each of the six homolog groups is challenged for achieving pairwise homologous synapsis in this karyotype, we typically observed just one or two intense H3K9me2 domains per nucleus (Figure 6A,B; 7B). Further, co-staining with SC markers revealed that while H3K9me2 localized to most regions lacking detectable SYP-1 in triploids (23/29), the H3K9me2 domains often failed to completely overlap such regions (16/29; Table S2). In addition, our analysis revealed another class of aberrant synapsis: axis segments associated with very low levels of SYP-1, which likely represent a subset of heterologously synapsed regions (Figure S7). These SYP-1-weak regions typically showed little or no H3K9me2 staining (28/37; Figure 6C, Table S2). These observations suggest that some of the many chromosomal regions experiencing synapsis problems in triploid hermaphrodites fail to acquire robust H3K9me2, and raise the possibility that incomplete marking of synapsis problems may be related to the dramatic extension of the chromosome mobilization phase in this karyotype.

### Males show a greater capacity to mark synapsis defects

We examined H3K9me2 and SUN-1 S8-Pi patterns in males with normal and altered karyotypes. Acquisition of H3K9me2 by the unsynapsed X during diploid male (1X:2A) spermatogenesis correlated temporally with loss of SUN-1 S8-Pi (Figure 7A), similar to triplo-X oogenesis. Triploid males (2X:3A) differed from triploid hermaphrodites, however, in that pachytene nuclei typically contained two to three major domains of H3K9me2 staining (Figure 7A, ii; 6B) – approximately one more than hermaphrodites despite the fact that they possess one less supernumerary chromosome. Further, while triploids of both sexes exhibited a similar range and degree of synapsis defects, males showed robust H3K9me2 marking of unsynapsed chromosome segments as well as a subset of (presumably heterologously) synapsed chromosome segments (Figure 7C). In addition, while the SUN-1 S8-Pi zone was extended relative to diploid males (Figure 7A, S8), we note that loss of SUN-1 S8-Pi took place well before the end of the pachytene region. The timing of meiotic progression differs between spermatogenesis and oogenesis, precluding a direct comparison of the two [26], [34]; however, this observation raises the possibility that triploid male germ cells resume meiotic progression more efficiently. Taken together, these data support the idea that synapsis-challenged chromosomes are more efficiently marked by the spermatogenic program than by the oogenic program.

### Diploid hermaphrodite germ cells display frequent synapsis imperfections marked by H3K9me2

**Figure 8:**
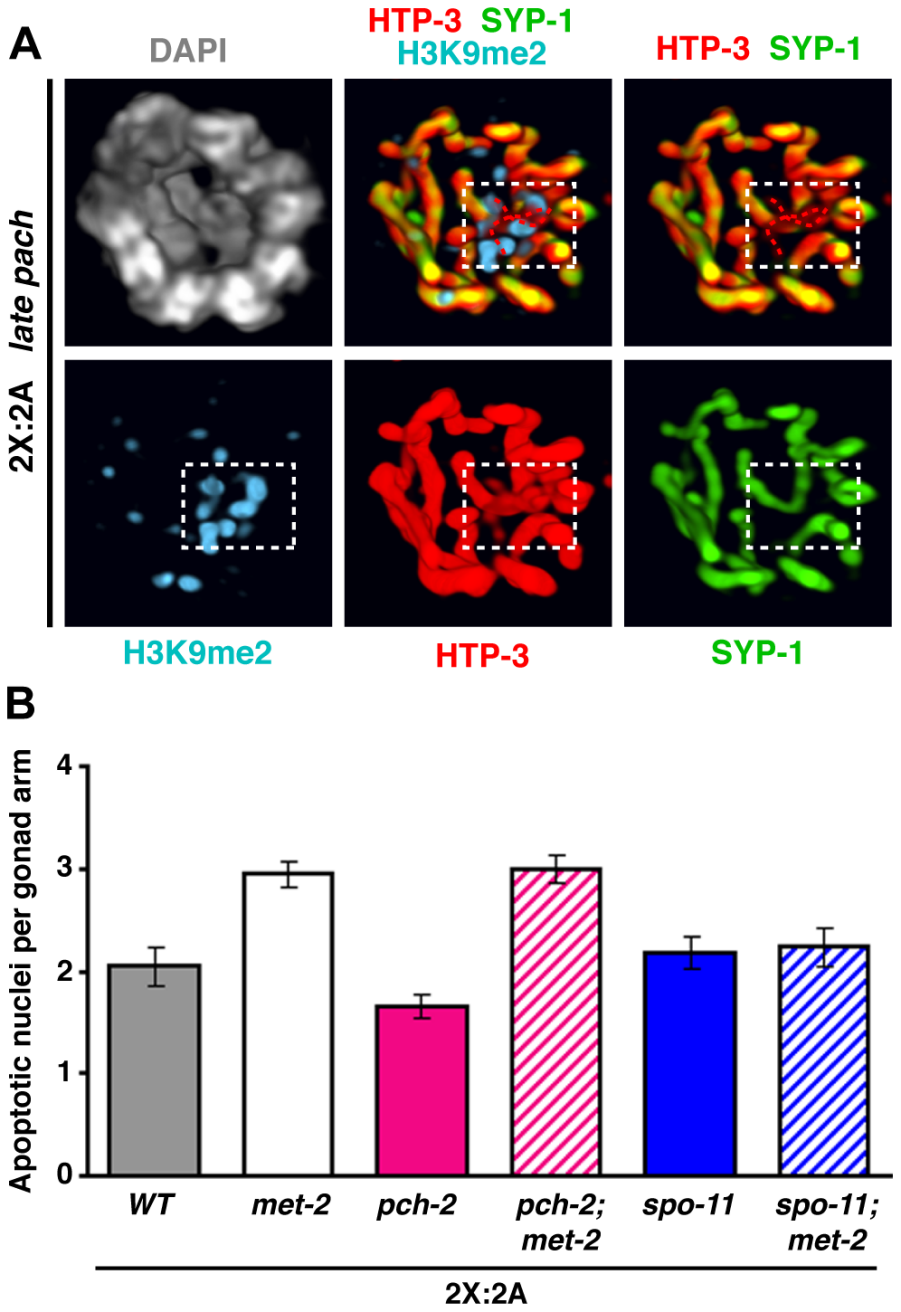
Detection of synapsis imperfections marked by H3K9me2 and elevated apoptosis upon depletion of MET-2 during normal diploid female meiosis. **A.** 3-D rendering of the bottom two-thirds of a late pachytene diploid nucleus stained for H3K9me2 (blue), HTP-3 (red), SYP-1 (green), and DAPI (gray). White box delineates a small internal H3K9me2 domain that marks terminally unsynapsed chromosome axes, highlighted by red dotted traces in merged images. **B.** Quantitaton of germ cell apoptosis in diploid hermaphrodites (24 h post-L4 at 20°C) of the indicated genotypes. Two-tailed Mann-Whitney tests indicate highly significant differences for *wild type* vs. *met-2* (P<0.0001), *pch-2* vs. *met-2; pch-2* (P<0.0001) and *met-2* vs. *met-2; spo-11* (P = 0.0049) comparisons, but not for *spo-11* vs. *met-2; spo-11* (P = 0.847) or *met-2* vs. *met-2; pch-2* (P = 0.961). Numbers of gonads scored: wild type, n = 71; *met-2*, n = 135; *pch-2*, n = 65; *met-2; pch-2*, n = 125; *spo-11*, n = 64; *met-2; spo-11*, n = 46.

Given the strong association between pachytene stage H3K9me2 domains and synapsis defects in meiotic mutants [32], [35] and in worms with altered karyotypes (this work), we closely examined H3K9me2 staining and synapsis status in diploid female germ cells, which display areas of H3K9me2 enrichment that are poorly understood. Rotation of 3-D images from the beginning of the late pachytene region, where specific signal is brightest, revealed concentration of H3K9me2 signal into small chromosome-associated domains in 23% of nuclei (32/142 nuclei from 5 germ lines) while the remaining nuclei showed dispersed signal (Figure 6B, i). 3-D rendering of individual diploid nuclei stained for H3K9me2 in combination with SC markers showed that these small H3K9me2 domains were typically located at the end of a SYP-1 stretch (31/35 domains from 5 germ lines) and oriented toward the nuclear interior (20/35). When both SYP-1 and HTP-3 were assessed, we detected small segments of HTP-3-positive chromosome axes lacking corresponding SYP-1 staining in 40% of H3K9me2 domain-containing nuclei (10/25 of such nuclei from 5 germ lines; Figure 8A), and all of these SYP-1-deficient segments colocalized with H3K9me2 domains. These observations indicate that small unsynapsed regions marked by H3K9me2 are present in a substantial percentage of normal diploid germ cells before large-scale desynapsis is observed.

In principle, small unsynapsed regions in approximately 9% of nuclei from the beginning of the late pachytene region could represent either unresolved synapsis defects or the onset of desynapsis. We reasoned that unresolved synapsis defects should be detected at the same or higher frequency earlier in meiotic prophase, whereas the converse should be true of desynapsing regions. Consistent with the former interpretation, examination of 3-D rendered images revealed small, internal, terminal regions of asynapsis in 16% of nuclei at the mid-pachytene stage (7/44 nuclei from 1 germ line). Although H3K9me2 staining was weaker at this stage, we detected signal at most unsynapsed regions (5/7), consistent with the idea that synapsis defects are marked by H3K9me2 around the time of exit from the chromosome mobilization phase in normal diploid meiocytes. We conclude that unresolved synapsis defects visible at the resolution of fluorescence microscopy are common during normal diploid meiosis, and are efficiently marked by H3K9me2.

### Histone methyltransferase MET-2 suppresses apoptosis during normal female meiosis

The MET-2 methyltransferase is responsible for the H3K9me2 chromatin modification in *C. elegans* germ cells [33]. MET-2 is required for transcriptional silencing of the partnerless X and for preventing activation of the recombination checkpoint in XO germ lines, implicating H3K9me2 in these processes [36], [37]; however, the modification did not appear to mediate silencing or checkpoint shielding for a pair of unsynapsed homologs. Thus, we tested whether MET-2 function and/or H3K9me2 acquisition by imperfectly synapsed regions could play a role in promoting meiotic progression and/or protecting meiocytes from apoptosis.

We found that a *met-2* mutation or depletion of MET-2 by RNAi caused loss of H3K9me2 without an accompanying extension of the SUN-1 S8-Pi zone in either diploid or triplo-X germ lines (Figure 6D and Materials and Methods). Thus, although H3K9me2 staining reveals that imperfectly synapsed regions acquire an altered character around the time of SUN-1 S8-Pi zone exit in these karyotypes, the modification itself is dispensable for exit from the mobilization phase and progression through meiosis.

However, loss of *met-2* function in diploid hermaphrodites did result in a significant elevation of germ cell apoptosis (Figure 8B; two-tailed P<0.0001, Mann-Whitney Test). This effect did not require PCH-2, suggesting that elevated apoptosis caused by loss of MET-2 does not reflect activation of the synapsis checkpoint. Instead, it was suppressed by loss of *spo-11* function, implying that elevated apoptosis in the *met-2* mutant reflects activation of the recombination checkpoint. These findings parallel the recent report that MET-2 prevents the partnerless X chromosome in XO germ cells from activating the recombination/DNA damage checkpoint [37]. Further, they are consistent with the possibility that MET-2 and H3K9me2 may play a role in protecting normal diploid meiocytes from undergoing apoptosis by inhibiting recombination intermediates associated with unsynapsed chromosome segments from activating the recombination checkpoint. However, we cannot rule out alternative interpretations, *e.g.*, that an altered distribution of DSBs or crossovers in a *met-2* mutant might somehow increase the likelihood of activating the recombination checkpoint.

The extent to which MET-2 might influence apoptosis levels in the presence of supernumerary chromosomes is less clear. Although *met-2 RNAi* did result in elevated apoptosis in diploid, triplo-X, triploid and tetraploid worms compared to empty vector controls (Figure S9), these results must be interpreted with caution since a recent report indicated that RNAi treatment *per se* can increase germ cell apoptosis [38]. Further, triplo-X *met-2(n4256)* mutant germ lines exhibited a very modest, albeit significant, elevation of apoptosis when compared with otherwise wild-type triplo-X controls. Thus, while MET-2 may help to limit checkpoint activation in the presence of an extra X chromosome, it may not be a major determinant of baseline apoptosis levels in this context. However, apoptosis levels were substantially elevated in triplo-X *spo-11* mutant germ lines (P<0.0001), suggesting that the atypical X-chromosome synapsis configurations observed in these worms are recognized as aberrant by the meiotic quality control machinery.

## Discussion

### Principles of pairing and synapsis illustrated by analysis of altered karyotypes

In this study, we challenged the meiotic program by manipulating karyotype in *C. elegans*. We began by defining pairing and synapsis phenotypes during trisomic and polyploid meiosis, taking advantage of high-resolution 3-D fluorescence imaging in the context of preserved nuclear architecture combined with an S-phase labeling method that affords both precise staging of meiocytes and delineation of the territory occupied by a specific chromosome group. While we found that synapsis was challenged in all altered karyotypes, overall we showed that tetraploids are able to achieve relatively complete, homologous synapsis, and triploids are more severely affected by synapsis failure than triplo-X worms.

This work complements classical microscopy studies of karyotype manipulation in a variety of organisms (for reviews, see [39], [40]), and highlights a set of general principles governing homolog pairing and synapsis. First, initial pairing and pre-synaptic alignment can take place among all homologous chromosomes present, even if greater than two; therefore, homolog recognition is not strictly pairwise in nature. Second, in all karyotypes, mature synapsis interactions tend to connect pairs of chromosome axes, indicating a preference for pairwise synapsis; however, supernumerary chromosomes appear to require more time to sort out pairwise interactions, and multi-partner synapsis and/or partner switches are sometimes observed. Third, synapsis failure is less prevalent than one might expect in karyotypes containing “odd” chromosomes that lack an exclusive, homologous pairing partner. Such chromosomes frequently engage in fold-back or non-homologous synapsis, indicating a drive to maximize synapsis interactions irrespective of homology [39], [41]. Fourth, our observations in triploids suggest that synapsis interactions may be remodeled over time. In combination with a number of previously documented phenomena such as SC “correction” in plants (reviewed in [42]), “synaptic adjustment” in a variety of organisms [39], [43], and incorporation of SC components throughout the pachytene stage in budding yeast [44], our work lends further credence to the view that the SC is a dynamic structure.

Unexpectedly, our work revealed a previously hidden influence of recombination on synapsis in *C. elegans*. Whereas recombination-based interactions are essential for normal pairing and/or synapsis in many organisms, it is well established that recombination is dispensable for homologous synapsis in *C. elegans* and Drosophila [29], [45], [46]. However, we found that in the context of triplo-X meiosis, *spo-11* status affects the ability to solidify the relationship between one pair of homologs while excluding a third, suggesting that recombination may contribute to synapsis fidelity even in an organism where recombination-independent mechanisms predominate. Interestingly, the apparent relationship between recombination and “exclusivity” in the formation of bivalents during *C. elegans* meiosis is reciprocal to that observed in *Bombyx mori*, where recombination occurs in males but not in females: recombination in tetraploid Bombyx males appears to lock in multi-partner relationships that are resolved into bivalent relationships in tetraploid females [47], [48], whereas recombination appears to promote two-partner exclusivity in triplo-X worms.

### Meiotic quality control mechanisms delay progression in response to synapsis challenges and eliminate defective meiocytes

Several quality control mechanisms have been identified that appear to contribute to meiotic success by ensuring that only meiocytes with properly synapsed chromosomes proceed to subsequent stages of meiosis. Analysis of the impact of karyotype alterations on meiotic progression and germ cell apoptosis provides insight into how these mechanisms operate. Our findings support and extend an existing model for a checkpoint-like mechanism that couples exit from the period of active chromosome movement with successful SC installation [19]. This model was initially proposed to explain the persistence of markers of chromosome mobilization into the late pachytene region (“extended TZ”) in mutants experiencing global asynapsis due to absence of SC precursors [17], and is supported by reports of similar responses to an unpaired and unsynapsed pair of chromosomes due to absence of specific PC proteins or deletion of the X-PCs [7], [8], [49]. Together, these observations imply that some aspect of unsynapsed chromosomes produces a signal that blocks exit from the chromosome mobilization phase.

Here, SUN-1 S8-Pi staining in trisomic and polyploid *C. elegans* indicates that odd numbers of chromosomes, which present synapsis challenges, also delay exit from the chromosome mobilization phase. While such a delay can be inferred from prior studies involving two unpaired and unsynapsed chromosomes [7], 8,49, prolonged mobilization in triplo-X germ lines clearly establishes that a single synapsis-challenged chromosome is sufficient to trigger this response. Further, the length of the delay appears to depend upon the number of chromosomes experiencing synapsis problems: triploids encountered a profound delay, whereas triplo-X germ cells resumed meiotic progression more quickly, and tetraploids were relatively unaffected. Together, these data suggest that the checkpoint-like coupling mechanism may delay exit from the mobilization phase in a manner that is sensitive to the dose of unsynapsed chromosomes.

Biological checkpoints typically function to prolong conditions necessary to resolve the lesions that trigger them. In this view, extension of the SUN-1 S8-Pi zone is a logical response to synapsis challenges because efficient SC assembly requires chromosome mobilization [9]. Although synapsis problems were enhanced in the context of karyotype alterations, we also found small unsynapsed regions in a significant fraction of wild-type pachytene meiocytes, suggesting that minor SC defects are common in diploids. In light of this finding, we propose that retention of chromosome mobilization during the early pachytene stage during normal meiosis may provide conditions necessary for resolution of synapsis problems that remain at a significant frequency after initial SC assembly.

A second class of mechanisms contributing to synapsis quality control consists of checkpoints that trigger apoptosis of late pachytene germ cells in response to asynapsis *per se*, or to recombination intermediates persisting at regions that are not synapsed with a homologous partner [24], [25]. Our data suggest that such checkpoints are not only relevant when meiosis is challenged by abnormal karyotype or mutation of meiotic machinery components, but also in the context of normal, diploid meiosis (see below).

During wild-type meiosis, stringent synapsis quality control can be achieved by sequential operation of these two types of mechanisms—prolonging conditions that promote resolution of synapsis problems, and then eliminating problems that fail to be corrected. We note that catastrophic synapsis problems (e.g., caused by absence of SC components [17] or triploidy (this work)) uncover limits to the capacity of synapsis quality control mechanisms: the chromosome mobilization phase can only be extended until the late pachytene region, and checkpoint-mediated apoptosis cannot cull all meiocytes. Under such circumstances, constraints on multiple meiotic processes are nevertheless ultimately lifted at the end of the pachytene region [19], [30]; this late-prophase lifting of constraints is proposed to serve as a fail-safe mechanism that safeguards the genome by facilitating DNA repair and restoration of genome integrity prior to chromosome segregation.

### “The perfect is the enemy of the good” -Voltaire: Evidence that saturable mechanisms for masking synapsis imperfections promote efficient meiosis

The purpose of pairing and synapsing homologous chromosomes during meiotic prophase is to enable the formation of crossover recombination events between homologs that underlie their ability to segregate at the meiosis I division. Given this raison d'etre, an effective scheme for promoting reproductive success would maximize assurance of crossover formation, rather than synapsis, as its outcome. Our analysis of meiosis in *C. elegans* with altered ploidy suggests that such a scheme does indeed operate. Specifically, our observations support a model in which the quality control mechanisms that respond to synapsis defects are counterbalanced by masking mechanisms that can “hide” limited defects (either asynapsed regions *per se*, or persistent recombination intermediates associated with such regions), allowing resumption of meiotic progression and evasion of checkpoint-mediated apoptosis.

Our data suggest that acquisition of the H3K9me2 chromatin modification by synapsis-challenged chromosomal regions reflects one aspect of masking, with the H3K9me2 mark identifying regions whose character has been altered by the meiotic program. We found that the unsynapsed or self-synapsed third X chromosome became marked with H3K9me2 in every triplo-X meiocyte, and this correlated temporally with loss of SUN-1 S8-Pi staining; a similar correlation was seen in tetraploids where synapsis-challenged regions also appeared to be efficiently marked. Observations in these karyotypes are therefore consistent with masking of synapsis problems relieving the block to meiotic progression.

Further, our data raise the possibility that this masking mechanism is saturable. In triploid hermaphrodite germ lines, where every homolog group experienced synapsis challenges, SUN-1 S8-Pi staining persisted until the end of the pachytene region. Our analysis indicated that some synapsis defects in triploid hermaphrodites were not marked with H3K9me2 (despite the fact that similar defects in triploid males acquired the mark robustly), suggesting that some defects remain unmasked in this karyotype. We propose that the amount of asynapsis in triploid hermaphrodites exceeds the system's capacity to “hide” problems, allowing signals that delay meiotic progression to persist. We further speculate that maximization of synapsis over time, irrespective of homology, may represent a separate means to hide chromosome regions without suitable pairing partners, thereby relieving the block to meiotic progression that unpartnered chromosome axes would otherwise present.

Taken together, our findings in trisomic and polyploid *C. elegans* support the existence of mechanisms that can mask synapsis problems up to a certain threshold. Importantly, we found that small synapsis defects in diploid pachytene germ cells were efficiently marked by H3K9me2, supporting the idea that these masking mechanisms also operate during the course of normal, wild-type meiosis. Although H3K9me2 serves as a useful marker for identifying masked regions with altered character, however, the modification itself participates in only a subset of the masking effects. Specifically, MET-2 and H3K9me2 do not appear to be required for the masking effect that enables meiotic progression. However, elevated apoptosis associated with loss of MET-2 function in diploids (this work) and XO germ cells [37] suggests that H3K9me2 is likely relevant for inhibiting checkpoint activation during normal diploid meiosis, presumably by preventing persistent recombination intermediates from triggering checkpoints. Together, our data suggest that the ability to limit a progression delay and the ability to prevent triggering of checkpoint-mediated apoptosis may operate at least partially through distinct mechanisms.

Our work raises the idea that “good enough”—rather than perfect—synapsis may be a preferable waypoint in the meiotic program. Partial homologous synapsis of all chromosome pairs should be sufficient to promote a single crossover on each, which is all that is required—and is in fact the normal state—in *C. elegans* meiosis. Examples of incomplete synapsis during normal meiosis have also been noted in other organisms (e.g., [50], [51]). Therefore, we propose that the ability to mask minor synapsis defects can provide an advantage by counterbalancing quality controls to ensure timely progression and survival of germ cells, and ultimately successful meiosis. It is well recognized that the meiotic program must employ mechanisms to accommodate asynapsis and lack of recombination for sex chromosome regions that lack a homologous partner [52]. We speculate that the saturable masking mechanism proposed here originated to handle the partnerless sex chromosome in males (1X:2A), and subsequently provided a benefit to reproductive fitness in both sexes.

Meiosis represents a multi-step biological program whose end goal is to segregate homologous chromosomes away from one another. The value of stringent quality control mechanisms monitoring intermediate steps required to achieve this goal (e.g., homolog pairing, synapsis, and crossover formation) is currently appreciated. Our work points out that such quality controls can be counterproductive, however, when perfection of an intermediate step is not an absolute requirement for achieving the desired end: “good enough” intermediates may be subjected to unnecessary delays or wasteful elimination. Therefore, by introducing the ability to hide minor defects unlikely to impact ultimate success, saturable masking mechanisms can provide a counterbalance to quality controls and improve the overall efficiency of the program. We suggest that the type of regulatory logic discussed here may be a widespread feature of biological circuits.

## Materials and Methods

### Strains and genetics

All *C. elegans* strains were cultivated at 20°C under standard conditions [53]. A mating stock of Bristol N2 provided the wild-type diploid background. Triplo-X worms are recognized by their semi-Dpy appearance [54] and were maintained by picking semi-Dpy L4s from the wild-type triplo-X “strain” AV494 at each generation. AV494 was generated by picking a spontaneous 3X:2A individual from CA257 (*him-8(tm611)* IV) which has a high degree of X chromosome nondisjunction, and backcrossing to N2 males to restore homozygosity for the wild-type *him-8* allele (confirmed by PCR) while maintaining a 3X:2A karyotype. The wild-type tetraploid strain SP346 [55] was maintained by picking Lon L4s every 1–2 generations. Most cytological analysis of tetraploids was conducted using worms that had been maintained on plates for less than 10 generations; accurate karyotype was confirmed by cytological phenotype at diakinesis (12 bivalents). Triploids (3X:3A hermaphrodites, and 2X:3A males) were generated as needed by mating N2 males (1X:2A) to Lon SP346 hermaphrodites (4X:4A) and were invariably the only large, healthy progeny produced in this cross; cytological phenotype at diakinesis was confirmed for 3X:3A hermaphrodites (6 bivalents+6 univalents).

Triplo-X “strains” AV785 and AV784, harboring the *met-2(n4256)* or *pch-2(tm1458)* mutations, respectively, were generated by crossing *met-2(n4256)/hT2[qIs48] (I;III)* or *pch-2(tm1458)* males with 3X:2A hermaphrodites from AV494. 3X:2A worms were plated individually at the F1 and F2 generations, and F2 plates homozygous for the *met-2* or *pch-2* mutation were identified by PCR.

Triplo-X “strain” AV783, heterozygous for *spo-11(me44)* and balancer chromosome *nT1[qIs51] (IV;V)*, was generated by crossing *spo-11(me44)/nT1[qIs51]* males with 3X:2A hermaphrodites from AV494. 3X:2A *+/nT1[qIs51]* cross progeny hermaphrodites (identified using a GFP marker associated with the balancer) were crossed with *spo-11(me44)/nT1[qIs51] (IV;V)* males. GFP+ 3X:2A hermaphrodites were plated individually in the next generation, and 3X:2A *spo-11(me44)/nT1[qIs51]* worms were identified by DAPI staining of their GFP- progeny: 3X:2A *spo-11(me44)* homozygotes were unambiguously identified based on the presence of 13 univalents in diakinesis-stage oocytes.

### S-phase labeling

For most experiments, S-phase labeling was performed by microinjection of fluorescent nucleotides as described [3], with the following modifications: young adult worms (24 hr post L4) were microinjected in both gonad arms with 1 mM Alexa Fluor 647-OBEA-dCTP (Invitrogen) or 0.1 mM Cy3-dCTP (GE Health Sciences); worms were recovered and maintained on food at 20°C until they were dissected and fixed for IF at the indicated time points post-injection. For the images shown in Figure 2A, S-phase labeling was performed by feeding EdU-labeled bacteria prepared as in [56]. Young adult worms (24 hr post L4) were placed on plates seeded with EdU-labeled bacteria and maintained at 20°C until dissection and fixation at the indicated time point; EdU was detected using the Click-iT EdU Alexa-555 Imaging kit (Invitrogen) as in [57]. Meiotic progression of labeled nuclei was indistinguishable between the two S-phase labeling techniques. To ensure consideration of a defined, synchronous population, a field of view containing the proximal front of labeled nuclei was imaged (see [3]).

### Immunofluorescence

All analysis was performed on approximately 24-h post-L4 adults, with the exception of S-phase-labeling experiments as described above. Dissection of gonads, fixation, immunostaining, and DAPI staining were performed essentially as in [19]. The following primary antibodies were used: guinea pig anti-HIM-8 (1∶500 [8]); rabbit anti-ZIM-3 (1∶2,000 [7]); chicken anti-HTP-3 (1∶250 [18]); rabbit anti-SYP-1 (1∶250 [17]); guinea pig anti-SUN-1 S8-Pi (1∶800 [11]); and mouse monoclonal anti-H3K9me2 (1∶400: abcam code: ab1220; lot: 629623). Secondary antibodies were Alexa Fluor 488, 555, and/or 647-conjugated goat antibodies directed against the appropriate species (1∶400; Invitrogen).

### Image collection, rendering and analysis

3-D images were collected as Z-stacks (0.1 or 0.2 µm step size) using a 60× NA 1.42 objective with 1.5× optivar or a 100× NA 1.40 objective on a DeltaVision widefield deconvolution microscopy system (Applied Precision) and deconvolved (except for Figure 3A) using softWoRx software. Additional 3-D images for the experiment presented in Figure 4 were collected on an OMX microscopy system (Applied Precision) in widefield mode using a 100× NA 1.4 objective, and deconvolved and corrected for registration. Contrast adjustments, 3-D cropping and image rendering (2-D maximum intensity projections or 3-D surface opacity renderings, including videos) were performed using the Volocity 5 software package (PerkinElmer). Final assembly of high-resolution, tiled germ lines, as well as minor contrast adjustments, were performed using Adobe Photoshop. All scoring of the attributes of S-phase labeled nuclei (Figure 2B,C; 3B; S3; S4) and triplo-X synapsis configurations (Figure 4), as well as evaluation of H3K9me2 staining with respect to synapsis status (Figure 6B; 7C; 8A; Table S2), was performed within Volocity using 3-D rendered images of individually cropped nuclei. For these experiments, nuclei were scored only when staining and resolution were of sufficient quality to unambiguously trace chromosome paths or SCs in 3-D rotations; approximately 3/4 of nuclei in the gonads scored met these criteria. For quantitation of synapsis configurations, we scored all nuclei showing label incorporation on the X chromosomes (or that were located within the specified zone) that met the inclusion criteria and were fully contained within the Z stacks. Length of the SUN-1 S8-Pi zone (Figure 5B, 5C, 6D, S8), SYTO 12 staining (Figure 8B, S6, S9), and numbers of meiotic zone nuclei (Table S1) were scored by eye on an AxioVision system (Zeiss).

### Scoring of germ cell apoptosis

Germ cell corpses were scored by SYTO 12 assay essentially as in [58] with the following modifications: 24-hour post-L4 adults were stained by picking into a 35 mM dilution of SYTO 12 (Molecular Probes) in M9 and incubating in the dark for 90 min at 20°C. Worms were destained by transferring to fresh seeded plates and incubating in the dark for 45 min at 20°C. Scoring was performed within a 45 min window on worms mounted in anesthetic mix in multiwell slides as in [59].

### *met-2* RNAi

RNAi was performed as in [3] with the following modifications: parents of the appropriate karyotype were placed on plates seeded with bacteria expressing dsRNA (empty vector L4440 or R05D3.11/*met-2*) from the Ahringer lab feeding RNAi library [60] at the L4 stage at 15°C and were allowed to produce progeny. For assessment of the SUN-1 S8-Pi zone, progeny were transferred to fresh RNAi plates at the L4 stage and incubated at 20°C for 24 hours prior to fixation for IF. The diploid strain AZ212 (*unc-119*(*ed3*) ruIs32 III [*pie-1*::GFP::H2B = *unc-119*(+)]), which exhibits increased sensitivity to RNAi [3], was used in the *met-2* RNAi/SUN-1 S8-Pi zone experiment. Length of the SUN-1 S8-Pi zone did not differ between the control and *met-2* RNAi for diploid AZ212 worms (58%+/−1.8% (n = 10 germ lines) vs. 55%+/−1.7% (n = 10) the meiotic zone, scored as in Figure 5) or triplo-X worms (72%+/−1.5% (n = 13) vs. 73%+/−1.5% (n = 16)). Efficient knockdown of *met-2* was assessed by IF for H3K9me2 in all RNAi experiments.

### Statistics

Statistical tests were performed using GraphPad InStat or Prism software.

## Supporting Information

Figure S1 ZIM-3 localizes to additional chromosome sites outside the Chromosome I and IV PCs. 3-D surface renderings of half the depth of two different early pachytene diploid nuclei stained for HTP-3 (pink) ZIM-3 (white) and DAPI (blue). Two major sites of ZIM-3 (white) staining of differing intensity (corresponding to the paired Chromosome I and IV PCs), as well as additional fainter ZIM-3 speckles elsewhere in the nucleus, are visible in each nucleus. Based on the reported number and distribution of ZIM-3 binding sites [61], we presume that the larger PC-associated site of ZIM-3 localized near the end of a chromosome axis corresponds to Chromosome I (orange arrows), and the smaller site at the very end of an axis corresponds to Chromosome IV (green arrows). In addition to the prominent PC staining, we detect ZIM-3 speckles localizing to secondary sites adjacent to the axes of chromosomes I and IV, similar to our recent report of localization of HIM-8 speckles to chromosome sites outside the X-PC [27]. Nucleus i highlights the typical localization of ZIM-3 speckles (orange carats) adjacent to the axis of presumptive Chromosome I (orange dotted trace). Nucleus ii highlights the typical localization of a ZIM-3 speckle (green carats) adjacent to the axis of presumptive Chromosome IV (green dotted trace).
(TIF)

Figure S2 Dynamics of PC pairing over time in altered karyotypes. **A.** Whole-mount germ lines of the indicated karyotypes immunostained for HIM-8 (red) and ZIM-3 (green) PC proteins. DAPI is shown in blue. Starting in the TZ of each germ line, a single HIM-8 focus and two ZIM-3 foci are seen in the large majority of nuclei in all karyotypes, consistent with primary PC pairing among groups of two, three or four homologous chromosomes. Around the early/mid-pachytene transition in the diploid, the intensity of ZIM-3 (and to a lesser extent, HIM-8) staining diminishes, as previously reported [7]. The location of this transition is shifted later in the triplo-X and especially the triploid, consistent with a delay in meiotic progression in karyotypes containing odd numbers of homologs. Following this transition in trisomic and polyploid meioses, HIM-8 foci sometimes appear as doublets, indicating loosening of the association among three or four X chromosomes as prophase progresses. Bar = 10 µm. **B.** Table of pairing frequencies for HIM-8 foci in nuclei with specific labeling of the X chromosomes at specific time points after S-phase labeling (10 h, 24 h, and 30 h, corresponding to late TZ, mid-pachytene and late pachytene stages in the diploid) in the indicated karyotypes. A single HIM-8 focus in the large majority of nuclei of all karyotypes at all time points indicates that two, three or four copies of the X chromosome can simultaneously pair at their PCs. The increased incidence of nuclei with >1 HIM-8 focus observed in tetraploids likely reflects the preference for pairwise synapsis, which may separate groups of four PCs that were originally paired.
(TIF)

Figure S3 Detailed analysis of X Chromosome territory configurations in late TZ region nuclei for each karyotype. 3-D rendered images of X chromosome territory configurations in all karyotypes at 10 h post-S phase, corresponding to the late TZ stage. Organization of images is analogous to Figure 2C. Upper panels show HIM-8 (green) and Cy5-dCTP label incorporated into the X chromosomes (red); lower panels also include DAPI (blue). In nuclei where all X-PCs were grouped together, which represented the large majority in all karyotypes, the morphology of X chromosome territories fell into two major classes: an extended territory or a compact mass. We speculate that these two morphologies reflect ongoing PC-led chromosome mobilization that can swirl the chromosomes into a ball or draw them out into an extended conformation. Only the “extended” class was informative for our purposes because it allowed us to assess whether the chromosomes diverge from one another or occupy a unitary domain consistent with alignment, although we note that chromosomes may be juxtaposed along their lengths in the compact conformation as well. In all karyotypes, paired and extended chromosomes typically exhibited a unitary appearance, consistent with close association along the length of the chromosomes as recently demonstrated in diploid nuclei of this stage using chromosome paints [27]. Rare trisomic and polyploid nuclei where X-PCs were separated into two groups are also depicted.
(TIF)

Figure S4 Triploid female germ cells transition toward exclusion of the third X chromosome and preferential pairwise synapsis by the end of meiotic prophase. **A.** Scoring of all classes of synapsis configurations observed for karyotypes with three X chromosomes at 24 hr (and 30 hr) post-S phase, corresponding to mid- (and late) pachytene stages in diploids. Most triploid nuclei at 24 hr post-S phase showed a unitary domain occupied by all three X chromosomes and containing one SC, in contrast with spatial exclusion of the third X chromosome from the synapsed pair in most triplo-X nuclei at this time point (two-sided P = 0.0003, Fisher's Exact Test). We observed a significant shift in triploids toward exclusion of the third X chromosome and emergence of pairwise synapsis by the 30 h time point (two-sided P = 0.0005, Fisher's Exact Test). Three categories of synapsis configurations were classified as fully pairwise: two X chromosomes fully synapsed plus the third X apparently (1) unsynapsed (as shown in Figure 3B, v), (2) synapsed with itself (as shown in Figure 3B, vi), or (3) heterologously synapsed with another non-X chromosome (see S4B, i, below). The total of these three categories is indicated in bold and presented adjacent to the category of nuclei in which a single SC was seen within one unitary X chromosome domain (as shown in Figure 3B, vii and viii, and S4B, ii, below). (n) = number of nuclei scored. **B.** Several prominent new classes of synapsis configurations were observed among three X chromosomes in triploids 30 h post-S phase labeling (red), as illustrated by 3-D surface renderings of individual nuclei stained for SYP-1 (white) and HIM-8 (green). i: Heterologous synapsis of the third X chromosome. While a pair of X chromosomes appears to share an SC (white dotted trace), the third X chromosome diverges from the pair beyond the X-PC (yellow arrow) and is associated with an adjacent SC (gray dotted trace) that is shared with another unlabeled chromosome. This SC appears to begin before the X joins it and to continue past the region in which the X is associated. ii: Three X chromosomes occupy a unitary domain that is associated with a single SC showing an unusual branched structure. Of the 18 triploid nuclei showing a single SC for three X chromosomes at this stage, six showed a branched SC, six showed an SC that contained a loop, and six showed a “normal” linear SC (as in Figure 3B, vii and viii). These observations reveal persistence of a minority of cases in which all three X chromosomes in triploid nuclei are connected by one SC (often of aberrant morphology), while the majority of triploid nuclei succeed in sorting their three X chromosomes into strictly pairwise interactions.
(TIF)

Figure S5 Detection of trivalent chromosomes at diakinesis confirms prior synapsis among three partner chromosomes. **A.** Upper: Image shows a projection of the full complement of chromosomes from a rare triploid diakinesis-oocyte containing five bivalents (“2”), five univalents (“1”), and one trivalent (“3”). (A typical triploid diakinesis figure contains six bivalents and six univalents.) Chromosomes are stained with DAPI (blue) and axis marker HTP-3 (red). Lower: rotated and magnified view of the trivalent, highlighting the double cruciform structure representing two chiasmata joining three chromosomes. A trivalent was verified in 2/69 triploid diakinesis figures analyzed. **B.** Schematic showing how two crossover recombination events can join three homologous chromosomes (depicted as purple, orange, and green pairs of sister chromatids) into a trivalent configuration. Since SC is required for crossover recombination, the fact that trivalents are detected implies that homologous synapsis can occur among three chromosomes. Three-way synapsis could be accomplished either by assembly of a single SC along the lengths of all three chromosomes, or by strictly pairwise synapsis at any given region with partner switches along the length of the chromosomes; our cytological methods did not distinguish among these possibilities. We note that the number of diakinesis trivalents detected may not accurately report the underlying frequency of three-way synapsis, as it is not known how crossover control would operate in the context of three-way synapsis.
(TIF)

Figure S6 Quantitation of germ cell apoptosis in worms with altered karyotypes. **A.** Worms were raised at 15°C on plates seeded with *E. coli* HT115 containing the empty RNAi vector L4440; worms were transferred to fresh plates at the late L4 stage and incubated at 15°C for an additional 24 h before processing for SYTO 12 staining. Raw numbers of apoptotic nuclei per gonad arm are plotted (mean +/− SEM). Two-tailed Mann-Whitney tests indicated that numbers of apoptotic nuclei per gonad arm were significantly elevated over diploid (2X:2A) in both 3X:2A (p<0.0001) and 3X:3A (p<0.0001) worms, but not in 4X:4A worms ( p = 0.859). Numbers of apoptotic nuclei per gonad arm were also significantly higher in 3X:3A worms than in 3X:2A worms (p<0.0001). Numbers of germ lines scored: 2X:2A, 101; 3X:2A, 59; 3X:3A, 56; 4X:4A, 34. **B.** Worms were raised at 15°C on plates seeded with *E. coli* OP50; worms were transferred to fresh plates at the late L4 stage and incubated at 20°C for an additional 24 h before processing for SYTO 12 staining. Raw numbers of apoptotic nuclei per gonad arm (mean +/− SEM) are plotted in the top graph; numbers of germ lines scored: 2X:2A, 116; 3X:2A, 97; 3X:3A, 107; 4X:4A, 83. Graphs below show the same data processed using two different normalization approaches attempting to account for the fact that the germ lines of worms with altered karyotypes contain different numbers of nuclei in their meiotic zones. To obtain the normalized values plotted in these graphs, we divided the observed mean numbers of corpses by a “sizing factor” for each karyotype, determined as follows (Table S1): Whole worms of each karyotype (24 h post L4 at 20°C) were subjected to ethanol fixation and DAPI staining as in [62]. Meiotic zone “length” was measured as the number of rows of nuclei from the first row in which two or more nuclei exhibited clustered chromosomes (transition zone) until the last pachytene row that contained multiple nuclei. The “width” was assessed at the mid-pachytene region by counting the number of rows spanning one face (top or bottom) of the tubular germ line. The sizing factors for each karyotype were calculated either as: [width] for indicated karyotype/[width] for the diploid (bottom left graph) or [length×width] for indicated karyotype/[length×width] for the diploid (bottom right graph). As only a subset of meiotic zone nuclei are developmentally competent to undergo apoptosis, it is unclear which of these normalization approaches is more appropriate; thus, both were included. Using the “width only” approach, normalized numbers were significantly elevated over diploid in 3X:2A (p = 0.005), 3X:3A (p = 0.0008) and 4X:4A worms ( p = 0.0002). Using the “length and width” approach, normalized numbers were significantly elevated over diploid in 3X:2A (p<0.0001), 3X:3A (p<0.0001) and 4X:4A worms (p<0.0001).
(TIF)

Figure S7 Evidence that weak SYP-1 tracks in triploid germ cells reflect heterologous synapsis. 3-D surface rendering of a triploid nucleus immunostained for SYP-1 (white) and HIM-8 (green) and counterstained with DAPI (blue) 30 h after S-phase labeling (red) of X chromosomes. An apparent pair of homologously synapsed X chromosomes is associated with a normal-intensity SYP-1 track (dense dotted trace). The third X chromosome is slightly separated from the pair at the X-PC and is associated with a faintly staining SYP-1 track (sparse dotted trace). The labeled X chromosome (red arrowhead) and an unlabeled chromosome identified by DAPI staining (blue arrowhead) run along opposite faces this faint SYP-1 track, indicating that this aberrant SC is assembled between heterologous segments. We also observe a class of heterologous synapsis characterized by normal-intensity SYP-1 tracks (see Figure S4B, nucleus i).
(TIF)

Figure S8 Triploid males display an extended SUN-1 S8-Pi zone. Quantitation of the percent of the meiotic prophase zone occupied by SUN-1 S8-Pi-positive nuclei in male diploids and triploids as assessed in immunostained whole mount germ lines. Scoring was performed as in Figure 5B, with the “meiotic prophase zone” defined as illustrated by the gray line in Figure 7A. Data are represented as mean +/− SEM. For each karyotype, number of germ lines scored: 1X:2A, 20; 2X:3A, 15.
(TIF)

Figure S9 Factors affecting apoptosis in the context of altered karyotypes. **A.** Quantitation of germ cell apoptosis in worms of the indicated karyotypes following *met-2 RNAi* treatment (by feeding). Experiments were conducted using 15°C conditions as described in Figure S6 and Materials and Methods. For all karyotypes, two-tailed Mann-Whitney tests indicated that numbers of apoptotic nuclei per gonad arm were significantly elevated in *met-2 RNAi* worms compared to controls: 2X:2A, p<0.0001; 3X:2A, p = 0.0021; 3X:3A, p<0.0001; 4X:4A, p = 0.0004. NOTE: While these results are consistent with MET-2 playing a role in limiting apoptosis, this conclusion must remain tentative in light of a recent report that *RNAi* (by injection) can cause elevated apoptosis [38]. Numbers of germ lines scored for controls: 2X:2A, 101; 3X:2A, 59; 3X:3A, 56; 4X:4A, 34. Numbers of germ lines scored for *met-2 RNAi*: 2X:2A, 97; 3X:2A, 55; 3X:3A, 51; 4X:4A, 55. **B.** Comparison of germ cell apoptosis levels for control 3X:2A worms (n = 147) and 3X:2A worms homozygous for the *met-2(n4256)* mutation (n = 113), showing a very modest but statistically significant increase in the *met-2* mutant background (p = 0.035). Worms were raised at 20°C and processed for SYTO 12 staining at 24 h post L4. **C.** Comparison of germ cell apoptosis levels for control 3X:2A worms (n = 100) and 3X:2A worms homozygous for either *pch-2(tm1458)* (n = 121) or *spo-11(me44)* (n = 37). Apoptosis levels were significantly elevated in the *spo-11* mutant background (p<0.0001) but not in the *pch-2* mutant background (p = 0.368). Worms were raised at 20°C and processed for SYTO 12 staining at 24 h post L4.
(TIF)

Table S1 Determination of sizing factors for normalization of apoptosis data.
(DOC)

Table S2 H3K9me2 marking of synapsis problems in triploid hermaphrodites is incomplete. A triploid hermaphrodite germ line was stained for HTP-3, SYP-1, and H3K9me2; twenty-five individual nuclei from the regions corresponding to the second half of mid-pachytene and the first half of late pachytene in diploids were analyzed. Nuclei were rendered in 3-D, and 66 synapsis defects were identified. These defects fell into two classes: 1) SYP-1 undetectable, where HTP-3 axis staining completely lacked corresponding SYP-1 central region staining; and 2) SYP-1 weak, where chromosome axes of normal intensity showed a substantial reduction in SYP-1 staining compared to surrounding SCs (see Figure 6C). For each defect, the proportion of the axial length surrounded by H3K9me2 staining was estimated and classified into one of the following four categories: none, less than half, equal to or greater than half, or essentially complete. For numbers cited in the main text, “some H3K9me2” included the three categories where the mark could be detected over at least part of the axis, “incomplete H3K9me2” included the three categories where the mark was not detected over the full length of the axis, and “little or no H3K9me2” included the categories in which the mark was detected over less than half or none of the axis. (n) = number of SC defects scored.
(DOC)

Video S1 3-D rotation of the triplo-X nucleus depicted in Figure 4A, i, showing an unsynapsed third X chromosome. DAPI (blue), HTP-3 (red), SYP-1 (green), and HIM-8 (white) are shown. In the starting and ending views, the X chromosome is positioned on the right side of the nucleus with the X-PC located approximately at 3 o'clock. The synapsed pair of X chromosomes is below the PC and the unsynapsed third X is above and to the left of it.
(MOV)

Video S2 3-D rotation of the triplo-X nucleus depicted in Figure 4A, ii, showing a third X chromosome with partial SYP-1 loading. DAPI (blue), HTP-3 (red), SYP-1 (green), and HIM-8 (white) are shown. In the starting and ending views, the X chromosome is positioned on the right side of the nucleus with the X-PC located approximately at 1 o'clock. The synapsed pair of X chromosomes extends below the PC and the third X is located above and to the left of it. Part of the HTP-3-stained axis of the third X chromosome is SYP-1-positive, while a SYP-1-free segment extends beyond this region (at approximately 12 o'clock), consistent with partial self-synapsis of the third X chromosome. (In this example and others, we noted that “self-synapsis” configurations sometimes appeared more complex than expected for simple folding back of the third X chromosome onto itself.)
(MOV)

Video S3 3-D rotation of the *spo-11* mutant triplo-X nucleus depicted in Figure 4B, showing a single SC associated with the territory corresponding to three X chromosomes. DAPI (blue), HTP-3 (red), SYP-1 (green), and HIM-8 (white) are shown. In the starting and ending views, the X chromosome is positioned on the right side of the nucleus with the X-PC located approximately at 12 o'clock and the SC extending below and to the right of it.
(MOV)
