# Supplementary figures and images for: Evidence That Masking of Synapsis Imperfections Counterbalances Quality Control to Promote Efficient Meiosis
Source: PLoS Genet. 2013 Dec 5;9(12):e1003963. doi: 10.1371/journal.pgen.1003963 (PMC3854781; doi:10.1371/journal.pgen.1003963)

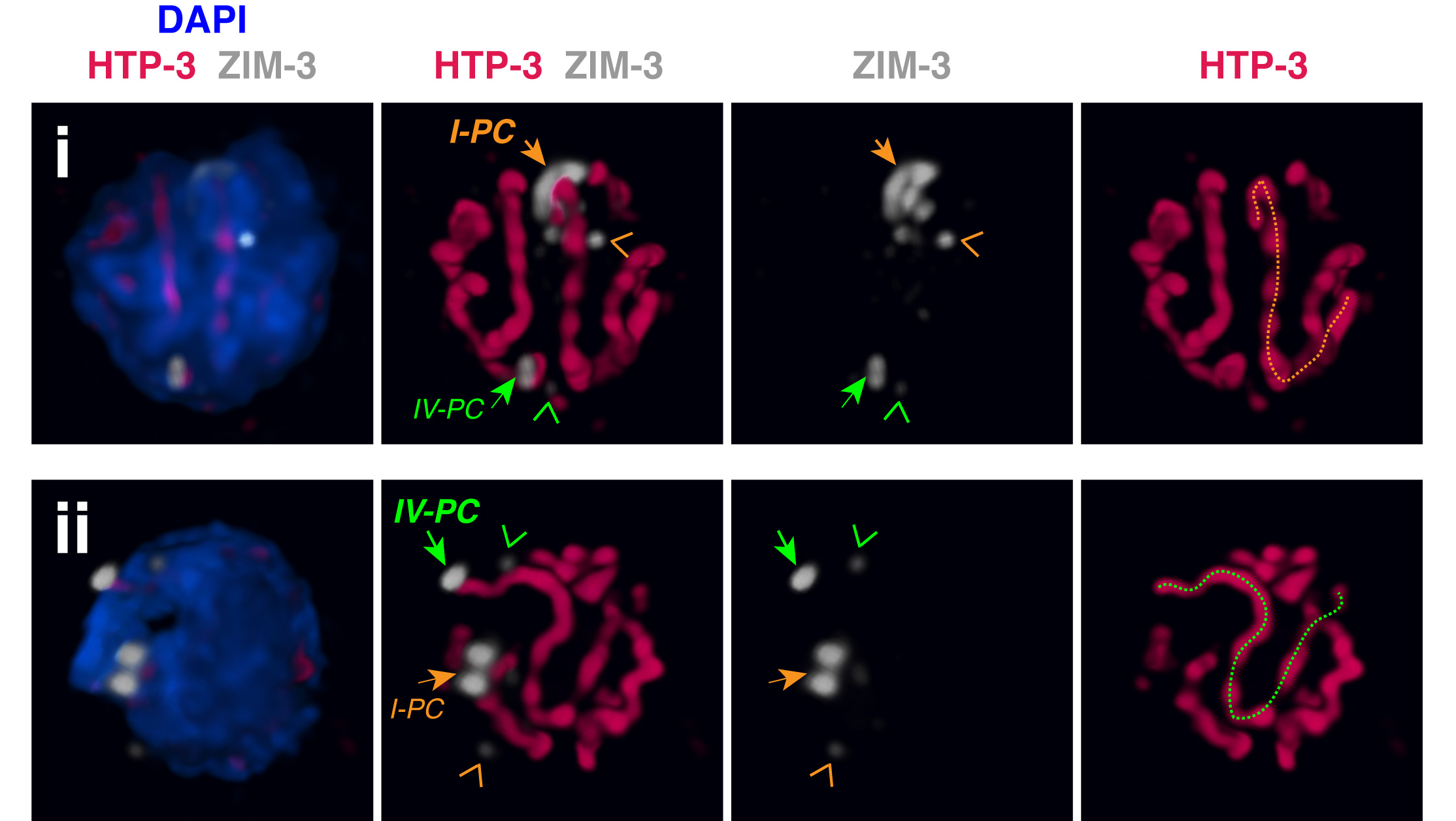

Supplement: Figure S1 — ZIM-3 localizes to additional chromosome sites outside the Chromosome I and IV PCs. 3-D surface renderings of half the depth of two different early pachytene diploid nuclei stained for HTP-3 (pink) ZIM-3 (white) and DAPI (blue). Two major sites of ZIM-3 (white) staining of differing intensity (corresponding to the paired Chromosome I and IV PCs), as well as additional fainter ZIM-3 speckles elsewhere in the nucleus, are visible in each nucleus. Based on the reported number and distribution of ZIM-3 binding sites [61], we presume that the larger PC-associated site of ZIM-3 localized near the end of a chromosome axis corresponds to Chromosome I (orange arrows), and the smaller site at the very end of an axis corresponds to Chromosome IV (green arrows). In addition to the prominent PC staining, we detect ZIM-3 speckles localizing to secondary sites adjacent to the axes of chromosomes I and IV, similar to our recent report of localization of HIM-8 speckles to chromosome sites outside the X-PC [27]. Nucleus i highlights the typical localization of ZIM-3 speckles (orange carats) adjacent to the axis of presumptive Chromosome I (orange dotted trace). Nucleus ii highlights the typical localization of a ZIM-3 speckle (green carats) adjacent to the axis of presumptive Chromosome IV (green dotted trace). (TIF) [file pgen.1003963.s001.tif]

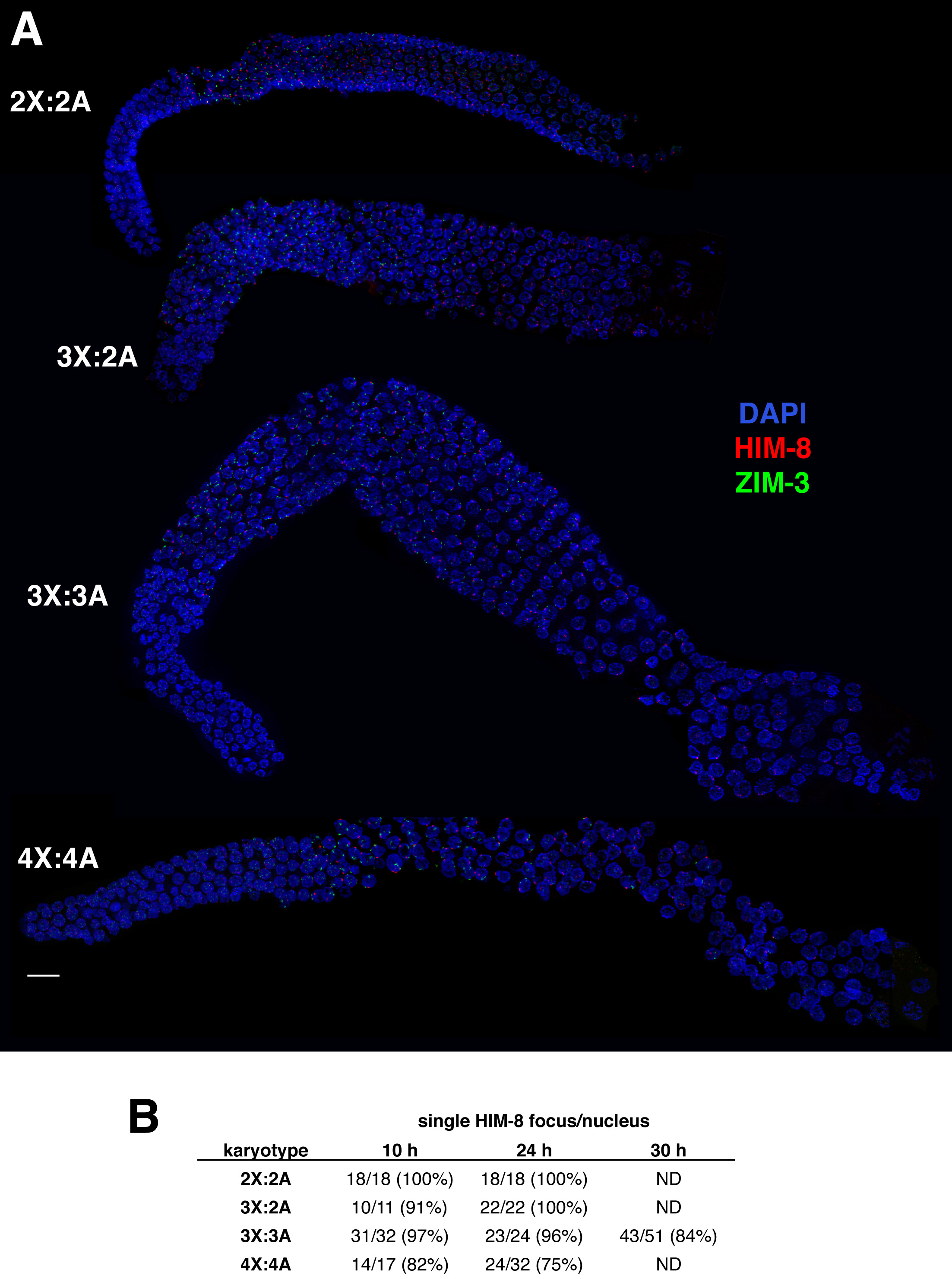

Supplement: Figure S2 — Dynamics of PC pairing over time in altered karyotypes. A. Whole-mount germ lines of the indicated karyotypes immunostained for HIM-8 (red) and ZIM-3 (green) PC proteins. DAPI is shown in blue. Starting in the TZ of each germ line, a single HIM-8 focus and two ZIM-3 foci are seen in the large majority of nuclei in all karyotypes, consistent with primary PC pairing among groups of two, three or four homologous chromosomes. Around the early/mid-pachytene transition in the diploid, the intensity of ZIM-3 (and to a lesser extent, HIM-8) staining diminishes, as previously reported [7]. The location of this transition is shifted later in the triplo-X and especially the triploid, consistent with a delay in meiotic progression in karyotypes containing odd numbers of homologs. Following this transition in trisomic and polyploid meioses, HIM-8 foci sometimes appear as doublets, indicating loosening of the association among three or four X chromosomes as prophase progresses. Bar = 10 µm. B. Table of pairing frequencies for HIM-8 foci in nuclei with specific labeling of the X chromosomes at specific time points after S-phase labeling (10 h, 24 h, and 30 h, corresponding to late TZ, mid-pachytene and late pachytene stages in the diploid) in the indicated karyotypes. A single HIM-8 focus in the large majority of nuclei of all karyotypes at all time points indicates that two, three or four copies of the X chromosome can simultaneously pair at their PCs. The increased incidence of nuclei with >1 HIM-8 focus observed in tetraploids likely reflects the preference for pairwise synapsis, which may separate groups of four PCs that were originally paired. (TIF) [file pgen.1003963.s002.tif]

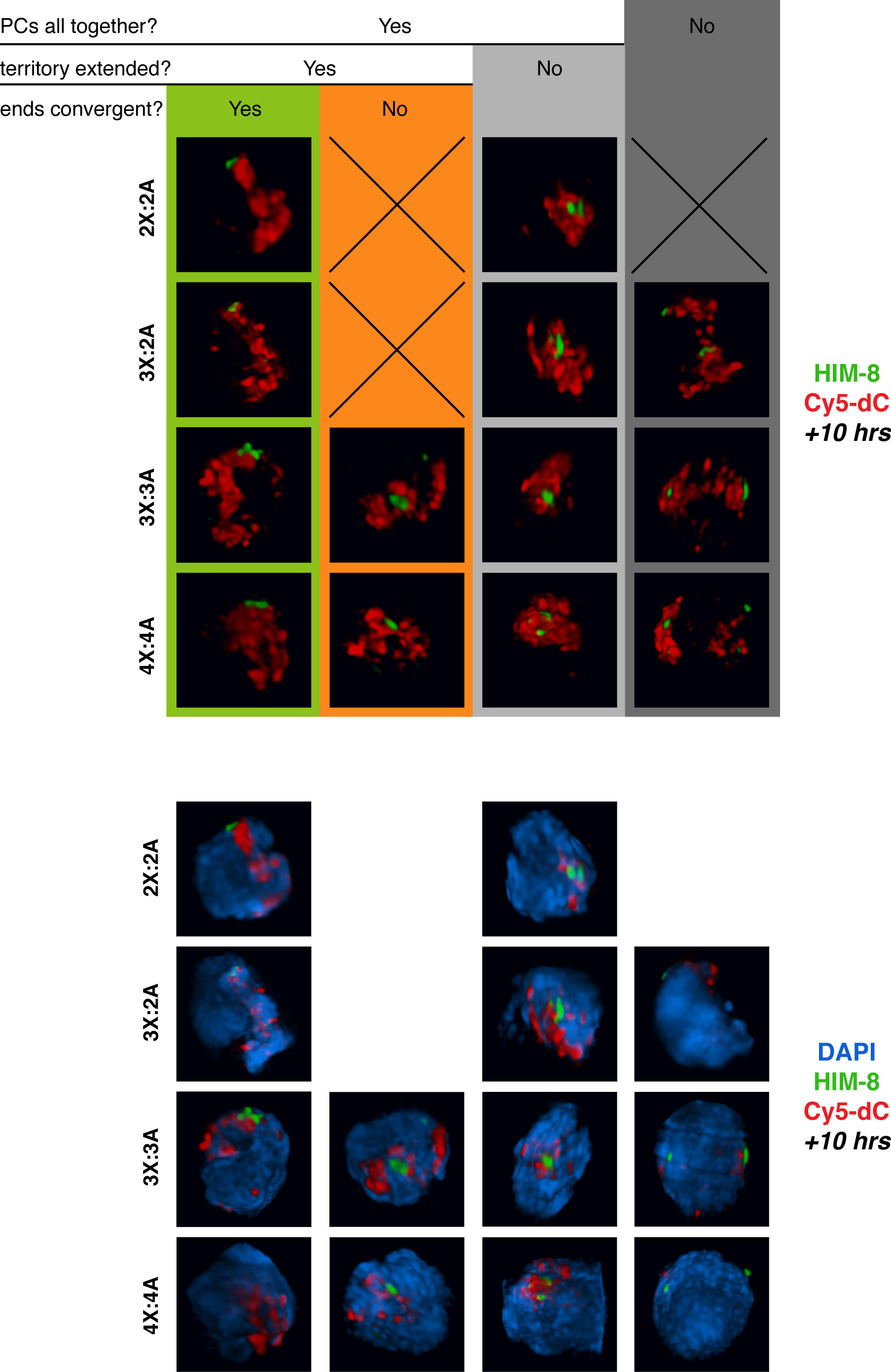

Supplement: Figure S3 — Detailed analysis of X Chromosome territory configurations in late TZ region nuclei for each karyotype. 3-D rendered images of X chromosome territory configurations in all karyotypes at 10 h post-S phase, corresponding to the late TZ stage. Organization of images is analogous to Figure 2C. Upper panels show HIM-8 (green) and Cy5-dCTP label incorporated into the X chromosomes (red); lower panels also include DAPI (blue). In nuclei where all X-PCs were grouped together, which represented the large majority in all karyotypes, the morphology of X chromosome territories fell into two major classes: an extended territory or a compact mass. We speculate that these two morphologies reflect ongoing PC-led chromosome mobilization that can swirl the chromosomes into a ball or draw them out into an extended conformation. Only the “extended” class was informative for our purposes because it allowed us to assess whether the chromosomes diverge from one another or occupy a unitary domain consistent with alignment, although we note that chromosomes may be juxtaposed along their lengths in the compact conformation as well. In all karyotypes, paired and extended chromosomes typically exhibited a unitary appearance, consistent with close association along the length of the chromosomes as recently demonstrated in diploid nuclei of this stage using chromosome paints [27]. Rare trisomic and polyploid nuclei where X-PCs were separated into two groups are also depicted. (TIF) [file pgen.1003963.s003.tif]

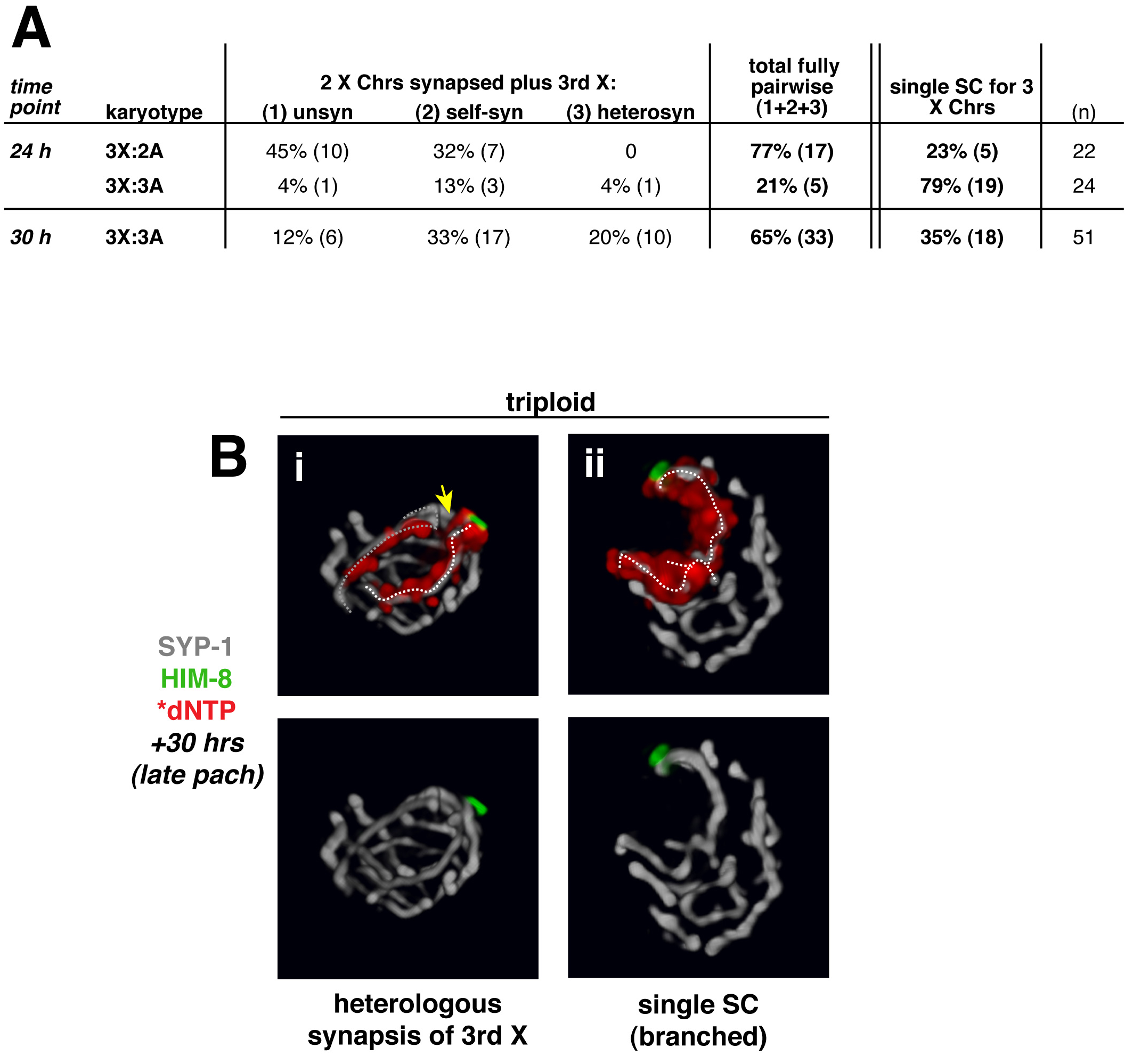

Supplement: Figure S4 — Triploid female germ cells transition toward exclusion of the third X chromosome and preferential pairwise synapsis by the end of meiotic prophase. A. Scoring of all classes of synapsis configurations observed for karyotypes with three X chromosomes at 24 hr (and 30 hr) post-S phase, corresponding to mid- (and late) pachytene stages in diploids. Most triploid nuclei at 24 hr post-S phase showed a unitary domain occupied by all three X chromosomes and containing one SC, in contrast with spatial exclusion of the third X chromosome from the synapsed pair in most triplo-X nuclei at this time point (two-sided P = 0.0003, Fisher's Exact Test). We observed a significant shift in triploids toward exclusion of the third X chromosome and emergence of pairwise synapsis by the 30 h time point (two-sided P = 0.0005, Fisher's Exact Test). Three categories of synapsis configurations were classified as fully pairwise: two X chromosomes fully synapsed plus the third X apparently (1) unsynapsed (as shown in Figure 3B, v), (2) synapsed with itself (as shown in Figure 3B, vi), or (3) heterologously synapsed with another non-X chromosome (see S4B, i, below). The total of these three categories is indicated in bold and presented adjacent to the category of nuclei in which a single SC was seen within one unitary X chromosome domain (as shown in Figure 3B, vii and viii, and S4B, ii, below). (n) = number of nuclei scored. B. Several prominent new classes of synapsis configurations were observed among three X chromosomes in triploids 30 h post-S phase labeling (red), as illustrated by 3-D surface renderings of individual nuclei stained for SYP-1 (white) and HIM-8 (green). i: Heterologous synapsis of the third X chromosome. While a pair of X chromosomes appears to share an SC (white dotted trace), the third X chromosome diverges from the pair beyond the X-PC (yellow arrow) and is associated with an adjacent SC (gray dotted trace) that is shared with another unlabeled chromosome. This SC appea [file pgen.1003963.s004.tif]

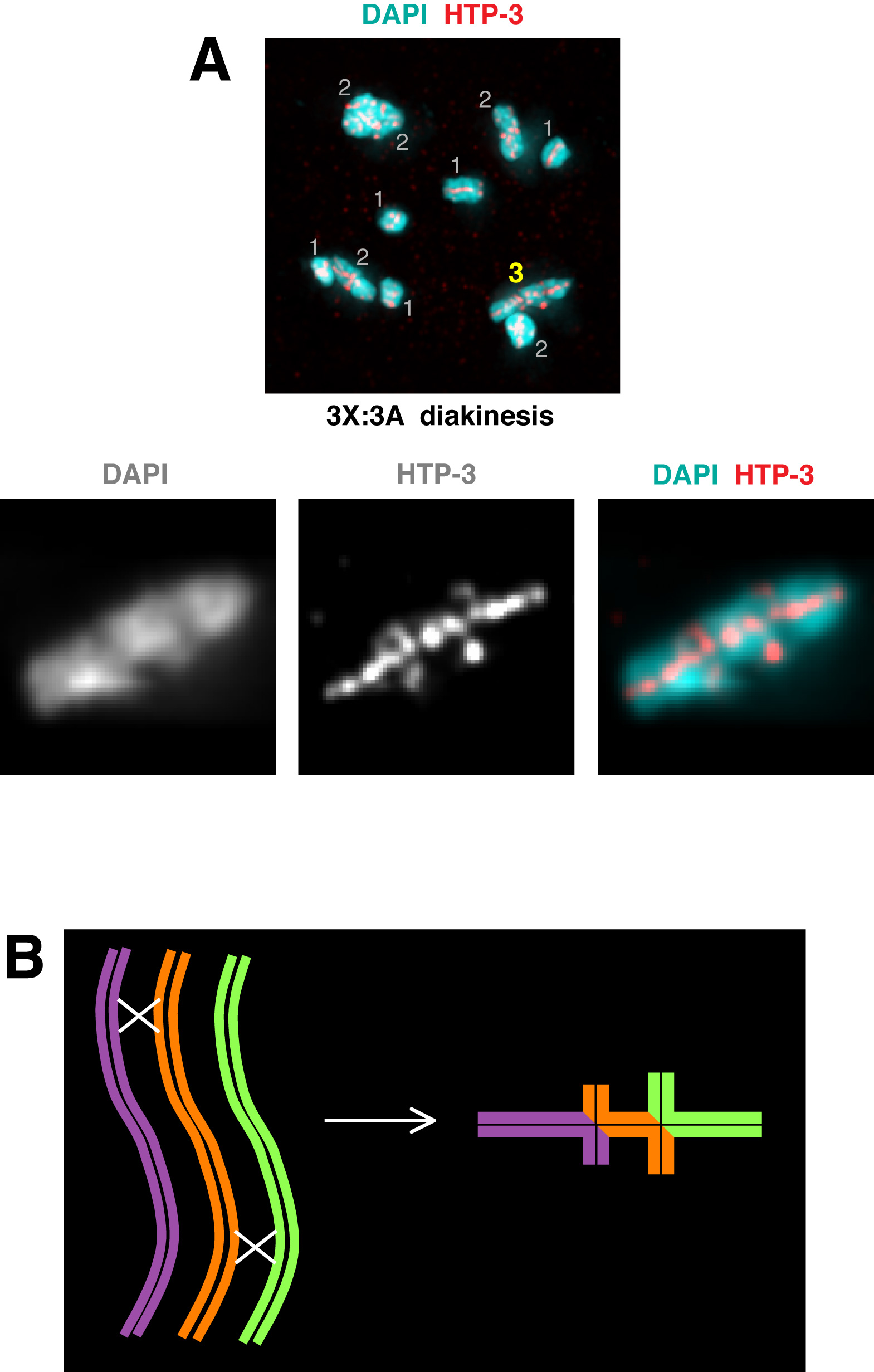

Supplement: Figure S5 — Detection of trivalent chromosomes at diakinesis confirms prior synapsis among three partner chromosomes. A. Upper: Image shows a projection of the full complement of chromosomes from a rare triploid diakinesis-oocyte containing five bivalents (“2”), five univalents (“1”), and one trivalent (“3”). (A typical triploid diakinesis figure contains six bivalents and six univalents.) Chromosomes are stained with DAPI (blue) and axis marker HTP-3 (red). Lower: rotated and magnified view of the trivalent, highlighting the double cruciform structure representing two chiasmata joining three chromosomes. A trivalent was verified in 2/69 triploid diakinesis figures analyzed. B. Schematic showing how two crossover recombination events can join three homologous chromosomes (depicted as purple, orange, and green pairs of sister chromatids) into a trivalent configuration. Since SC is required for crossover recombination, the fact that trivalents are detected implies that homologous synapsis can occur among three chromosomes. Three-way synapsis could be accomplished either by assembly of a single SC along the lengths of all three chromosomes, or by strictly pairwise synapsis at any given region with partner switches along the length of the chromosomes; our cytological methods did not distinguish among these possibilities. We note that the number of diakinesis trivalents detected may not accurately report the underlying frequency of three-way synapsis, as it is not known how crossover control would operate in the context of three-way synapsis. (TIF) [file pgen.1003963.s005.tif]

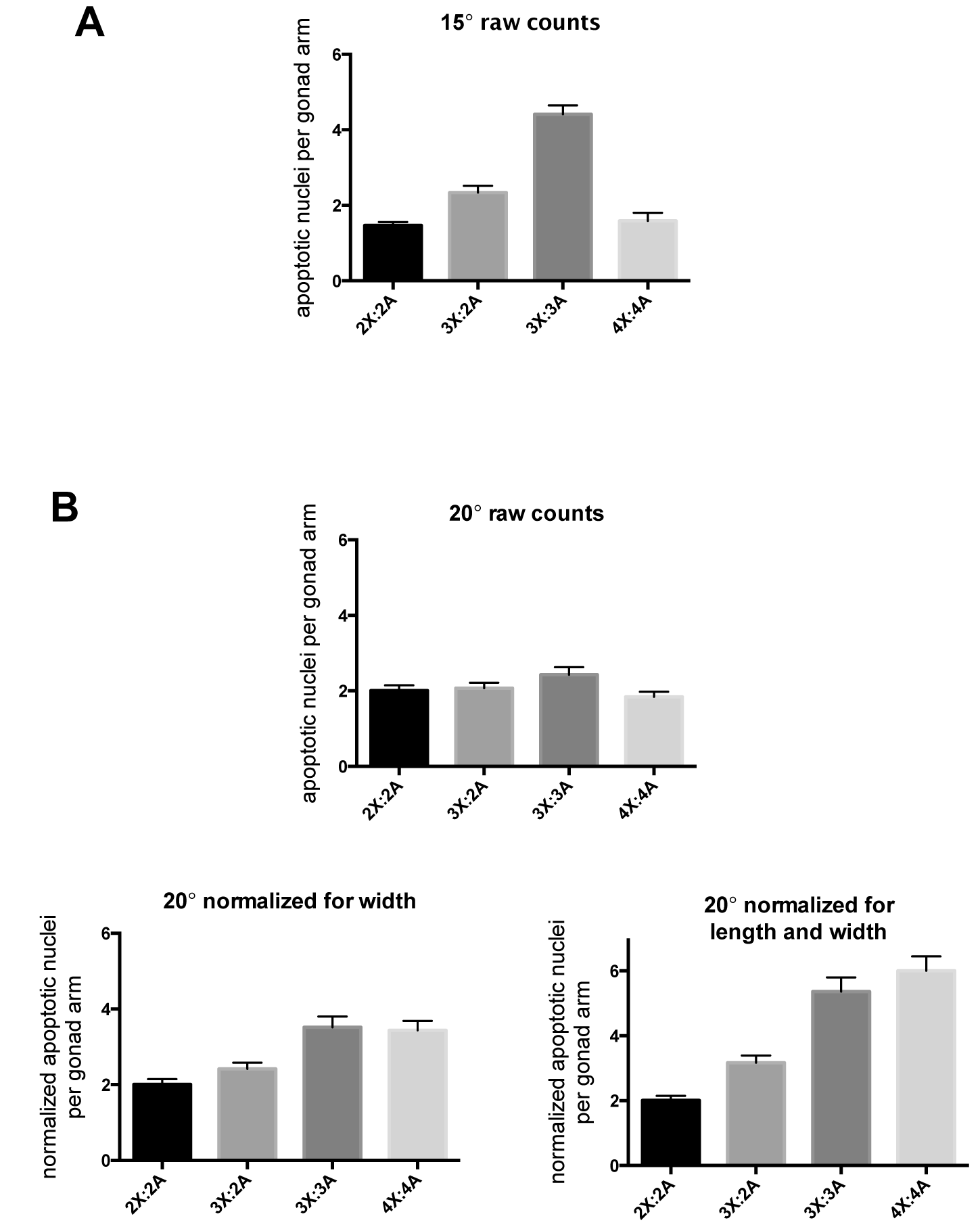

Supplement: Figure S6 — Quantitation of germ cell apoptosis in worms with altered karyotypes. A. Worms were raised at 15°C on plates seeded with E. coli HT115 containing the empty RNAi vector L4440; worms were transferred to fresh plates at the late L4 stage and incubated at 15°C for an additional 24 h before processing for SYTO 12 staining. Raw numbers of apoptotic nuclei per gonad arm are plotted (mean +/− SEM). Two-tailed Mann-Whitney tests indicated that numbers of apoptotic nuclei per gonad arm were significantly elevated over diploid (2X:2A) in both 3X:2A (p<0.0001) and 3X:3A (p<0.0001) worms, but not in 4X:4A worms ( p = 0.859). Numbers of apoptotic nuclei per gonad arm were also significantly higher in 3X:3A worms than in 3X:2A worms (p<0.0001). Numbers of germ lines scored: 2X:2A, 101; 3X:2A, 59; 3X:3A, 56; 4X:4A, 34. B. Worms were raised at 15°C on plates seeded with E. coli OP50; worms were transferred to fresh plates at the late L4 stage and incubated at 20°C for an additional 24 h before processing for SYTO 12 staining. Raw numbers of apoptotic nuclei per gonad arm (mean +/− SEM) are plotted in the top graph; numbers of germ lines scored: 2X:2A, 116; 3X:2A, 97; 3X:3A, 107; 4X:4A, 83. Graphs below show the same data processed using two different normalization approaches attempting to account for the fact that the germ lines of worms with altered karyotypes contain different numbers of nuclei in their meiotic zones. To obtain the normalized values plotted in these graphs, we divided the observed mean numbers of corpses by a “sizing factor” for each karyotype, determined as follows (Table S1): Whole worms of each karyotype (24 h post L4 at 20°C) were subjected to ethanol fixation and DAPI staining as in [62]. Meiotic zone “length” was measured as the number of rows of nuclei from the first row in which two or more nuclei exhibited clustered chromosomes (transition zone) until the last pachytene row that contained multiple nuclei. The “width” was assessed at the mid-pachytene regi [file pgen.1003963.s006.tif]

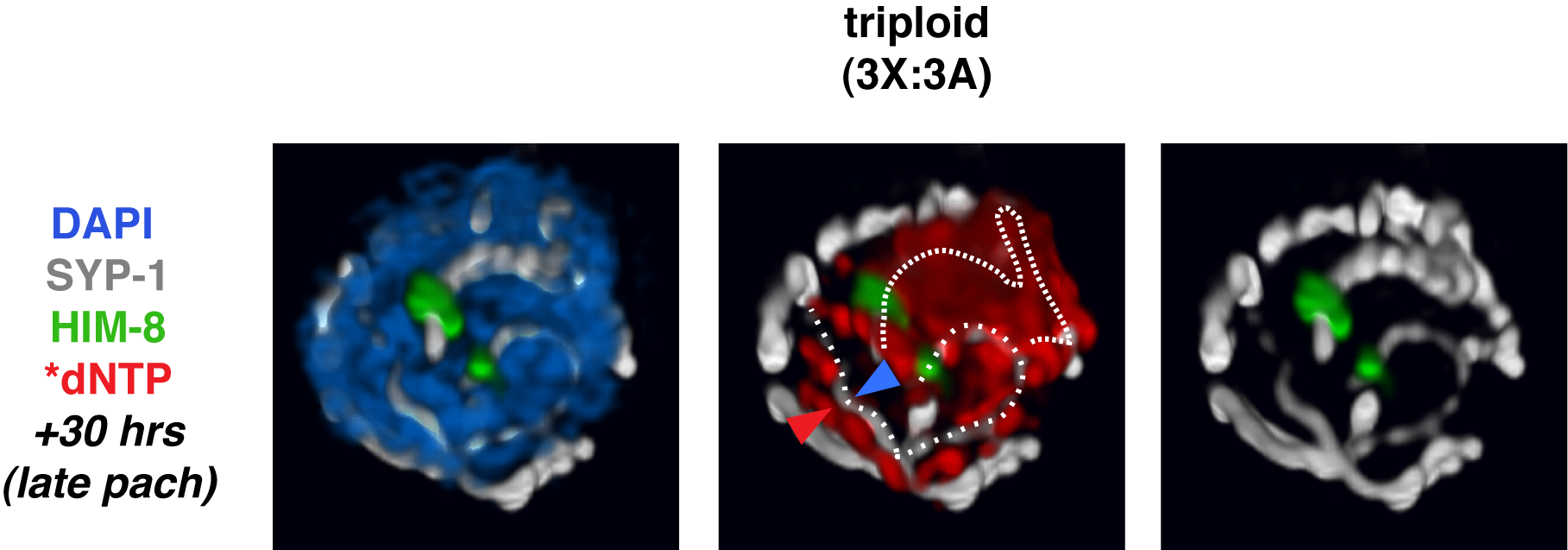

Supplement: Figure S7 — Evidence that weak SYP-1 tracks in triploid germ cells reflect heterologous synapsis. 3-D surface rendering of a triploid nucleus immunostained for SYP-1 (white) and HIM-8 (green) and counterstained with DAPI (blue) 30 h after S-phase labeling (red) of X chromosomes. An apparent pair of homologously synapsed X chromosomes is associated with a normal-intensity SYP-1 track (dense dotted trace). The third X chromosome is slightly separated from the pair at the X-PC and is associated with a faintly staining SYP-1 track (sparse dotted trace). The labeled X chromosome (red arrowhead) and an unlabeled chromosome identified by DAPI staining (blue arrowhead) run along opposite faces this faint SYP-1 track, indicating that this aberrant SC is assembled between heterologous segments. We also observe a class of heterologous synapsis characterized by normal-intensity SYP-1 tracks (see Figure S4B, nucleus i). (TIF) [file pgen.1003963.s007.tif]

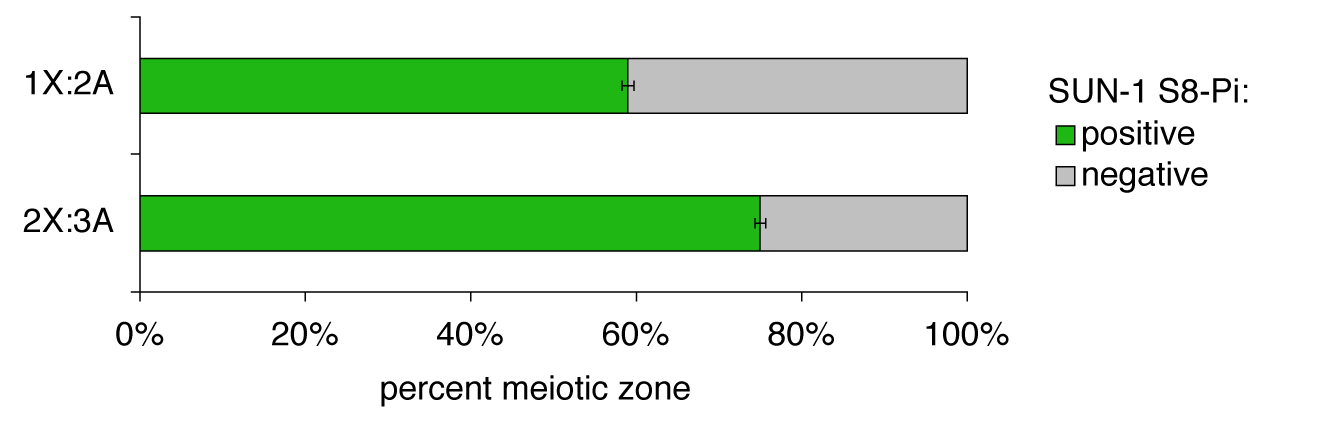

Supplement: Figure S8 — Triploid males display an extended SUN-1 S8-Pi zone. Quantitation of the percent of the meiotic prophase zone occupied by SUN-1 S8-Pi-positive nuclei in male diploids and triploids as assessed in immunostained whole mount germ lines. Scoring was performed as in Figure 5B, with the “meiotic prophase zone” defined as illustrated by the gray line in Figure 7A. Data are represented as mean +/− SEM. For each karyotype, number of germ lines scored: 1X:2A, 20; 2X:3A, 15. (TIF) [file pgen.1003963.s008.tif]

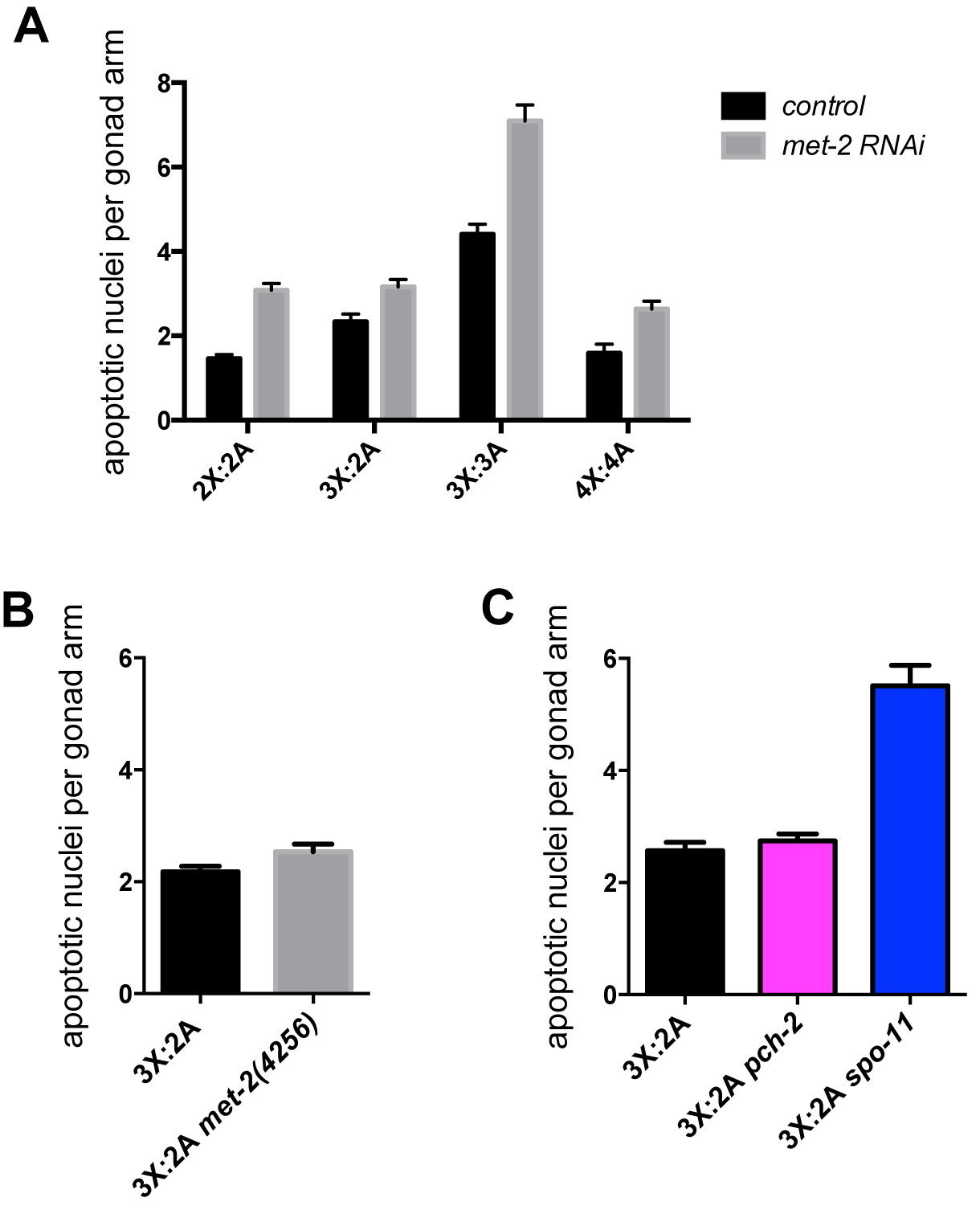

Supplement: Figure S9 — Factors affecting apoptosis in the context of altered karyotypes. A. Quantitation of germ cell apoptosis in worms of the indicated karyotypes following met-2 RNAi treatment (by feeding). Experiments were conducted using 15°C conditions as described in Figure S6 and Materials and Methods. For all karyotypes, two-tailed Mann-Whitney tests indicated that numbers of apoptotic nuclei per gonad arm were significantly elevated in met-2 RNAi worms compared to controls: 2X:2A, p<0.0001; 3X:2A, p = 0.0021; 3X:3A, p<0.0001; 4X:4A, p = 0.0004. NOTE: While these results are consistent with MET-2 playing a role in limiting apoptosis, this conclusion must remain tentative in light of a recent report that RNAi (by injection) can cause elevated apoptosis [38]. Numbers of germ lines scored for controls: 2X:2A, 101; 3X:2A, 59; 3X:3A, 56; 4X:4A, 34. Numbers of germ lines scored for met-2 RNAi: 2X:2A, 97; 3X:2A, 55; 3X:3A, 51; 4X:4A, 55. B. Comparison of germ cell apoptosis levels for control 3X:2A worms (n = 147) and 3X:2A worms homozygous for the met-2(n4256) mutation (n = 113), showing a very modest but statistically significant increase in the met-2 mutant background (p = 0.035). Worms were raised at 20°C and processed for SYTO 12 staining at 24 h post L4. C. Comparison of germ cell apoptosis levels for control 3X:2A worms (n = 100) and 3X:2A worms homozygous for either pch-2(tm1458) (n = 121) or spo-11(me44) (n = 37). Apoptosis levels were significantly elevated in the spo-11 mutant background (p<0.0001) but not in the pch-2 mutant background (p = 0.368). Worms were raised at 20°C and processed for SYTO 12 staining at 24 h post L4. (TIF) [file pgen.1003963.s009.tif]
