# Supplementary material for: Evidence That Masking of Synapsis Imperfections Counterbalances Quality Control to Promote Efficient Meiosis
Source: PLoS Genet. 2013 Dec 5;9(12):e1003963. doi: 10.1371/journal.pgen.1003963 (PMC3854781; doi:10.1371/journal.pgen.1003963)
Supplement: Table S1 — Determination of sizing factors for normalization of apoptosis data. (DOC) [file pgen.1003963.s010.doc]

Table S1. Determination of sizing factors for normalization of apoptosis data.

|  | **meiotic zone length (rows, +s.d.)** | **mid-pachytene width (rows, +s.d.)** | **sizing factor**  **(width only)** | **sizing factor**  **(length and width)** | **number of gonads scored** |
| --- | --- | --- | --- | --- | --- |
| **2X:2A** | **43.9** +3.2 | **9.2** +1.0 | **1** | **1** | 16 |
| **3X:2A** | **33.5** +4.4 | **7.9** +1.2 | **.86** | **.65** | 17 |
| **3X:3A** | **28.8** +3.6 | **6.4** +1.8 | **.69** | **.45** | 20 |
| **4X:4A** | **25.1** +3.5 | **4.9** +0.8 | **.54** | **.31** | 14 |
