# Supplementary material for: Evidence That Masking of Synapsis Imperfections Counterbalances Quality Control to Promote Efficient Meiosis
Source: PLoS Genet. 2013 Dec 5;9(12):e1003963. doi: 10.1371/journal.pgen.1003963 (PMC3854781; doi:10.1371/journal.pgen.1003963)
Supplement: Table S2 — H3K9me2 marking of synapsis problems in triploid hermaphrodites is incomplete. A triploid hermaphrodite germ line was stained for HTP-3, SYP-1, and H3K9me2; twenty-five individual nuclei from the regions corresponding to the second half of mid-pachytene and the first half of late pachytene in diploids were analyzed. Nuclei were rendered in 3-D, and 66 synapsis defects were identified. These defects fell into two classes: 1) SYP-1 undetectable, where HTP-3 axis staining completely lacked corresponding SYP-1 central region staining; and 2) SYP-1 weak, where chromosome axes of normal intensity showed a substantial reduction in SYP-1 staining compared to surrounding SCs (see Figure 6C). For each defect, the proportion of the axial length surrounded by H3K9me2 staining was estimated and classified into one of the following four categories: none, less than half, equal to or greater than half, or essentially complete. For numbers cited in the main text, “some H3K9me2” included the three categories where the mark could be detected over at least part of the axis, “incomplete H3K9me2” included the three categories where the mark was not detected over the full length of the axis, and “little or no H3K9me2” included the categories in which the mark was detected over less than half or none of the axis. (n) = number of SC defects scored. (DOC) [file pgen.1003963.s011.doc]

**Table S2.** **H3K9me2 marking of synapsis problems in triploid hermaphrodites is incomplete.**

|  | **H3K9me2 staining** | | | |  |
| --- | --- | --- | --- | --- | --- |
|  | **none** | **<half** | **>half** | **~complete** | *(n)* |
| **SYP-1 undetectable** | 10% (3) | 10% (3) | 34% (10) | 45% (13) | *29* |
| **SYP-1 weak** | 30% (11) | 46% (17) | 19% (7) | 5% (2) | *37* |
| **Total** | **21% (14)** | **30% (20)** | **26% (17)** | **23% (15)** | *66* |
